# *O*-glycosylation of the transcription factor SPATULA promotes style development in *Arabidopsis*

**DOI:** 10.1038/s41477-023-01617-4

**Published:** 2024-01-26

**Authors:** Yuxiang Jiang, Seamus Curran-French, Samuel W. H. Koh, Iqra Jamil, Benguo Gu, Luca Argirò, Sergio G. Lopez, Carlo Martins, Gerhard Saalbach, Laila Moubayidin

**Affiliations:** 1 Department of Cell and Developmental Biology, John Innes Centre, Norwich, UK; 2 Department of Molecular Microbiology, John Innes Centre, Norwich, UK

**Keywords:** Plant morphogenesis, Plant reproduction

## Abstract

*O*-linked β-*N*-acetylglucosamine (*O*-GlcNAc) and *O*-fucose are two sugar-based post-translational modifications whose mechanistic role in plant signalling and transcriptional regulation is still largely unknown. Here we investigated how two *O*-glycosyltransferase enzymes of *Arabidopsis thaliana*, SPINDLY (SPY) and SECRET AGENT (SEC), promote the activity of the basic helix–loop–helix transcription factor SPATULA (SPT) during morphogenesis of the plant female reproductive organ apex, the style. SPY and SEC modify amino-terminal residues of SPT in vivo and in vitro by attaching *O*-fucose and *O*-GlcNAc, respectively. This post-translational regulation does not impact SPT homo- and heterodimerization events, although it enhances the affinity of SPT for the kinase *PINOID* gene locus and its transcriptional repression. Our findings offer a mechanistic example of the effect of *O*-GlcNAc and *O*-fucose on the activity of a plant transcription factor and reveal previously unrecognized roles for SEC and SPY in orchestrating style elongation and shape.

## Abstract (web summary)

The *Arabidopsis* *O*-glycosyltransferases SEC and SPY modify the transcription factor SPT and synergistically promote style elongation. Specific SPT residues modified by *O*-GlcNAc and *O*-fucose are essential for style radialization.

## Main

The gynoecium, a sophisticated organ within the flower, ensures fertilization and seed production in angiosperms. Depending on plant species and/or developmental window, gynoecium shape may greatly vary to complement its function^1,2^. Of notable importance is the morphological diversity observed at the apical-distal end of the gynoecium, encompassing the style and stigma. This region serves as the landing site for pollen, where germination occurs, and subsequently facilitates the pollen’s journey to fertilize the ovules^2^. Both plant fitness and seed production are thus intricately linked to the correct development of the style and stigma. This developmental process is highly dynamic, and it is underpinned by a complex molecular orchestration that operates in an intertwined manner^3,4^.

Among the transcription factors (TFs) presiding over gynoecium development, the activity of SPATULA (SPT)—a key regulator of medial tissue identity and style morphogenesis^5^—is pivotal in orchestrating auxin accumulation as well as coordinating the medio-lateral and adaxial–abaxial polarity axis^6–8^ and repressing cytokinin (CK)-mediated cell-proliferation input at the gynoecium apex^9^. SPT forms homo- and heterodimers with specific TF partners to modulate several aspects of style development^4,9–12^. Accordingly, *SPT* loss-of-function mutants fail to form a fused, radially symmetric style at the gynoecium apex^5^ (producing a so-called split style), and this phenotype is exacerbated by mutations in other basic helix–loop–helix (bHLH) TFs such as INDEHISCENT (IND)^13^ and HECATE 1, 2 and 3 (HEC1,2,3)^8,11^ as well as members of the NGATHA protein family^4^.

Because of its stable spatiotemporal expression in the apical-medial tissues during gynoecium development^5,8^, we hypothesized that the dynamic activity of SPT required for style patterning may be regulated at the protein level. Interestingly, a proteomic investigation of *Arabidopsis* flower proteins showed that SPT is post-translationally modified by *O*-linked β-*N*-acetylglucosamine (*O*-GlcNAc)^13^.

*O*-GlcNAc is a post-translational modification (PTM) associated with key cellular processes and stress responses in both animals and plants^14–18^. In both kingdoms, *O*-GlcNAc is attached to a plethora of proteins encompassing various cellular functions, among which TFs are highly abundant^19–21^. *O*-GlcNAc decoration of target proteins leads to the modulation of their stability, cellular localization, protein–protein interactions, transcriptional activity and other characteristics^20,22^. Mechanistic studies in the animal field have shown that *O*-GlcNAc modifications can impact TF activity by changing, for example, their DNA-affinity binding, their ability to bind transcriptional co-activators/co-repressors and/or their domain function^20,22–25^.

Despite its importance across kingdoms and biological scales, the translation of *O*-GlcNAc modification into specific protein functions remains predominantly uncharted territory. This knowledge gap is particularly pronounced in the plant kingdom, where the mechanistic understanding of how sugar-based PTMs influence the activity of TFs is notably lacking. The modification of SPT by *O*-GlcNAc might thus provide a rapid, adaptable mechanism to intricately regulate SPT activity during gynoecium development.

In *Arabidopsis*, the enzyme SECRET AGENT (SEC) catalyses the addition of *O*-GlcNAc from UDP–GlcNAc to Ser and/or Thr residues of acceptor-substrate proteins^18^. A functionally related enzyme encoded by the gene locus *SPINDLY* (*SPY*)^26^ functions as an *O*-fucosyltransferase (POFUT), attaching monofucose (*O*-fucose) to target substrates^26,27^. Notably, while single *sec* and *spy* mutant alleles are perfectly viable in *Arabidopsis*, *sec* *spy* double mutants are embryo lethal (similar to *ogt* mutants in animals^28,29^), highlighting the synergistic and fundamental importance of both enzymes for plant development^30^.

Examples of mechanistic outcomes resulting from the SEC-mediated *O-*glycosylation of their acceptor targets include alteration in protein–protein interactions of the RNA-binding protein TaGRP2 during wheat vernalization^31^ and of the gibberellin repressor RGA in *Arabidopsis*^18^. Given that RGA does not directly bind to DNA, it has been proposed that *O*-GlcNAc modification of this transcriptional repressor could elicit a conformational change in its protein structure. This structural alteration, in turn, would negatively impact the binding of RGA to its protein interactors, thereby facilitating the progression of gene expression^18,26^. In contrast, the modification of RGA by *O*-fucose would trigger an open conformation that retains the binding of RGA to its protein interactors, leading to repression of the downstream signalling pathways, opposite to the effect of *O*-GlcNAc^18,26^.

Our investigation allows a pioneering understanding of the functional implications of *O*-GlcNAc modifications for plant TF activity. By means of genetic, molecular, biochemical and proteomic experiments, we herein demonstrate a role for SEC and SPY in style development via post-translational regulation of SPT activity. We demonstrate that SPT directly interacts with SEC and SPY, which modify the amino terminus of SPT by *O*-GlcNAc and *O*-fucose, respectively, both in vivo and in vitro. Moreover, via a genetic complementation assay, we provide evidence that specific modified residues located in two N-terminal peptides are essential for SPT function in vivo, accounting for style morphogenesis. Furthermore, we show that both enzymes enhance the transcriptional activity and DNA-binding affinity of SPT to the *PINOID* (*PID*) gene locus^32^, but do not impact SPT nuclear localization, protein stability or dimerization events with itself, IND or HEC1. Accordingly, the genetic epistasis analysis between SEC/SPY and SPT, coupled with the style morphological defects displayed by the inducible *SEC* knockdown in a *spy* mutant background (*SEC RNAi* *spy-3*), corroborates a model in which SPT activity is primed by SEC and SPY transferases to fine-tune its downstream control of the hormonal balance and support style development.

Our findings provide a mechanistic insight into the role of *O*-GlcNAc and *O*-fucose PTMs in the activity of a plant TF, characterize a post-embryonic target modified synergistically by both SEC and SPY during style development, and reinforce the importance of the upstream regulation mediated by these sugar-based PTMs on key TFs important for organ morphogenesis in multicellular organisms.

## Results

### SPT is modified by *O*-GlcNAc and *O*-fucose via SEC and SPY

**Fig. 1:**
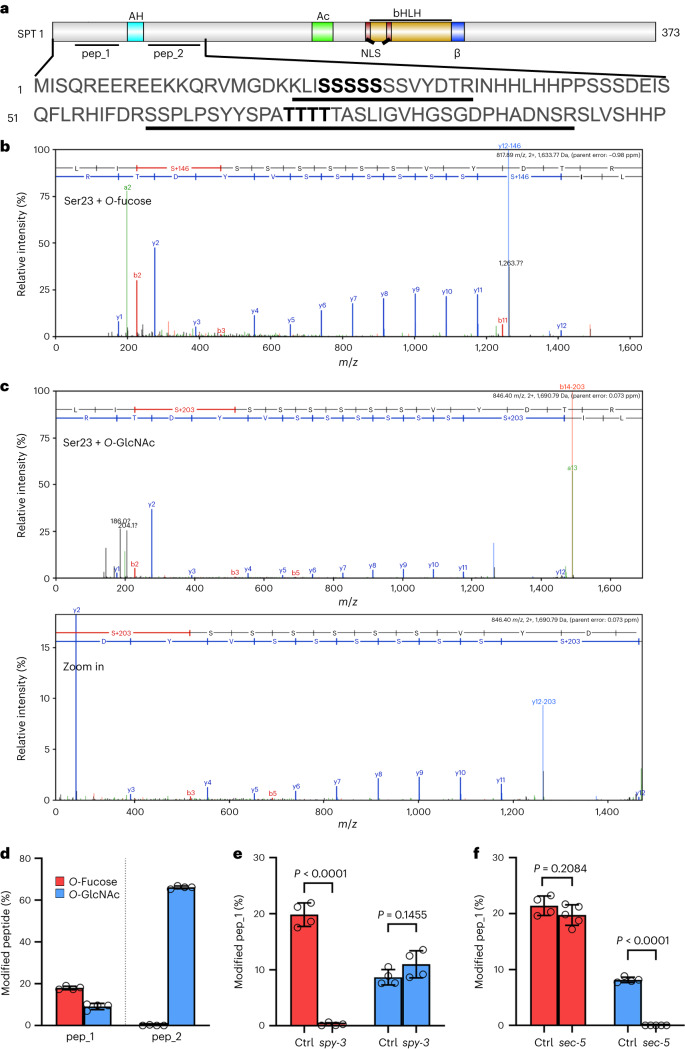
SPT is modified in vivo by both *O-*fucose and *O-*GlcNAc via SPY and SEC activity, respectively. **a**, Illustration of the full-length SPT protein and its domains (NLS, nuclear localization signal; β, β strand^36^). The positions of the two peptides (pep_1 and pep_2, black lines) targeted by *O-*glycosyl PTMs are displayed flanking the AH domain. The SPT N-terminal amino acid sequence is displayed, including pep_1 and pep_2 (underlined sequences). Residues in bold indicate regions for which *O-*glycosyl modifications have been detected. **b**,**c**, Representative HCD spectra of SPT pep_1 indicating modification by *O-*fucose (**b**) and *O-*GlcNAc (**c**) (bottom in **c**, zoom-in spectrum) on residue Ser23. Note the presence of a complete and unmodified y-ion series up to y11 and a shift in mass of the y12 ion. Similar spectra are provided in Extended Data Fig. 3. **d**–**f**, Quantification of the percentage of *O-*fucosylation (red bars) and *O*-GlcNacylation (blue bars) recovered on both SPT peptides in the *spt-12*/*SPT*::*SPT–sYFP* complementation line inflorescences (*n* = 4 biological independent samples for each peptide) (**d**) and quantification of the same modifications recovered on pep_1 in the *spy-3* (*n* = 4 for both the control (ctrl) and *spy-3*) (**e**) and *sec-5* (*n* = 4 for ctrl and *n* = 5 for *sec-5*) (**f**) mutant backgrounds. The values shown are means ± s.d. Significant differences and *P* values are indicated in the graphs following two-tailed Student’s *t*-tests. Source data

**Extended Data Fig. 1:**
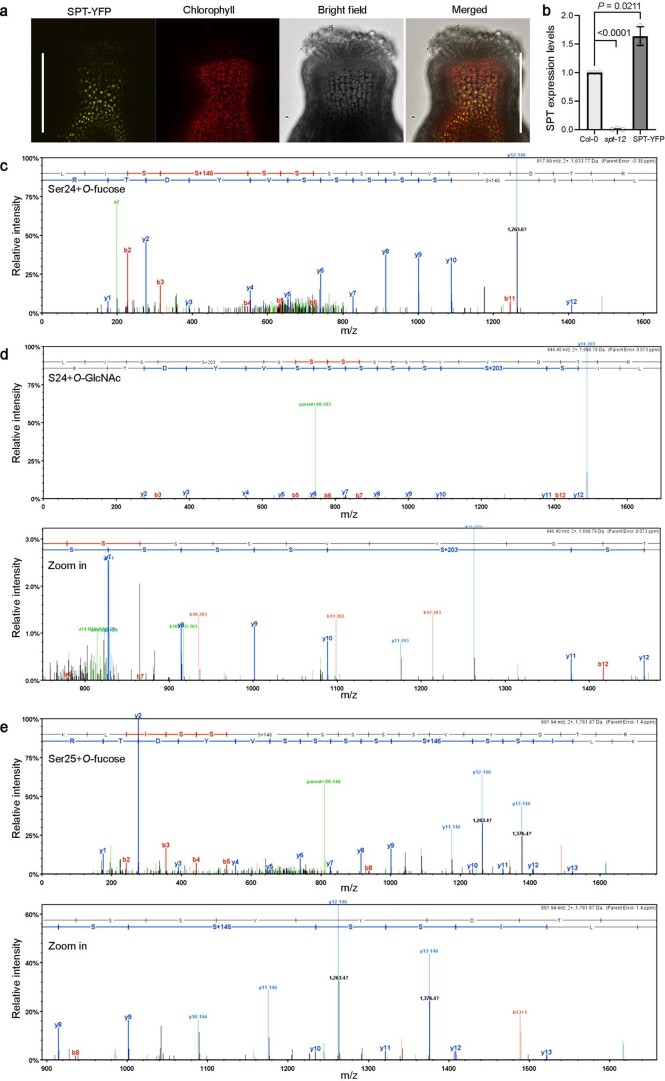
*In vivo* proteomic analysis of *spt*/*SPT-YFP* complementation line inflorescences. **a**, Confocal image (split-channels) of a stage-12 gynoecium apex of the *spt-12/SPT::SPT-sYFP* complementation line (hereafter use SPT-YFP to represent the translated protein product), which fully restored the split-style phenotype of *spt-12* mutant. Note, expression of nuclear YFP signal is shown in the style. Scale bars indicate 500 μm. **b**, Quantification of *SPT* expression levels by qRT-PCR in inflorescences of Col-0, *spt-12* and *spt-12/SPT::SPT-sYFP* complementation line. Values shown are means ± SD from three biological repeats. *P* values indicate two-tailed *t* test results. **c-e**, Representative HCD spectra of SPT pep_1 indicating modifications of residue Ser24 by both *O-*fucose (c) and *O-*GlcNAc (d) (zoom-in spectrum, lower panel) and residue Ser25 modified by *O-*fucose (e) (zoom-in spectrum, lower panel). Source data

To understand whether a post-translation mechanism based on *O*-glycosylation could preside over SPT-mediated control of style development, we performed higher-energy collisional dissociation (HCD) and electron transfer higher-energy collision dissociation (EThcD) fragmentation mass spectrometry (MS/MS) analyses on nuclear extracts from fully rescued *spt-12/SPT*::*SPT*–*sYFP* complementation line inflorescences (Extended Data Fig. 1a,b). The analyses identified several Ser and Thr residues at the N terminus of SPT that were modified by two *O*-linked sugar moieties, *O*-fucose and *O*-GlcNAc. In vivo, the HCD spectra indicated that both sugar groups can be localized on Ser23 and Ser24 (recovered in peptide_1, LISSSSSSSVYDTR), while Ser25 was found modified by *O*-fucose (Fig. 1a–c and Extended Data Fig. 1c–e). EThcD analysis of the same peptide showed a wider distribution of modifications; it indicated that the glycans could be attached to Ser23, Ser24 and Ser25 and also showed that Ser26 and Ser27 can be modified (Supplementary Table 1).

The HCD spectra corresponding to the second peptide of interest, initiating with Ser60 (peptide_2: SSPLPSYYSPATTTTTASLIGVHGSGDPHADNSR), did not provide sufficient information for the precise localization of the glycan modifications. However, the observed precursor mass strongly suggests the presence of a modification consistent with *O*-GlcNAc (Fig. 1a). Furthermore, all EThcD spectra obtained for peptide_2 consistently indicated that one of the Thr71-to-Thr74 residues was the site of the modification (Supplementary Table 2). These findings align with a prior investigation performed on *O*-GlcNAc-modified *Arabidopsis* flower proteins^21^.

The quantification of both modifications on the two N-terminal peptides of SPT was then carried out by normalizing the abundances of each modified peptide version to the total sum of the abundances of all versions (modified and unmodified) of the same peptide. The levels of *O*-fucose modification were higher than those of *O*-GlcNAc on peptide_1, while on peptide_2, *O*-GlcNAcylation was conspicuously elevated and *O*-fucosylation was barely detected (Fig. 1d).

**Extended Data Fig. 2:**
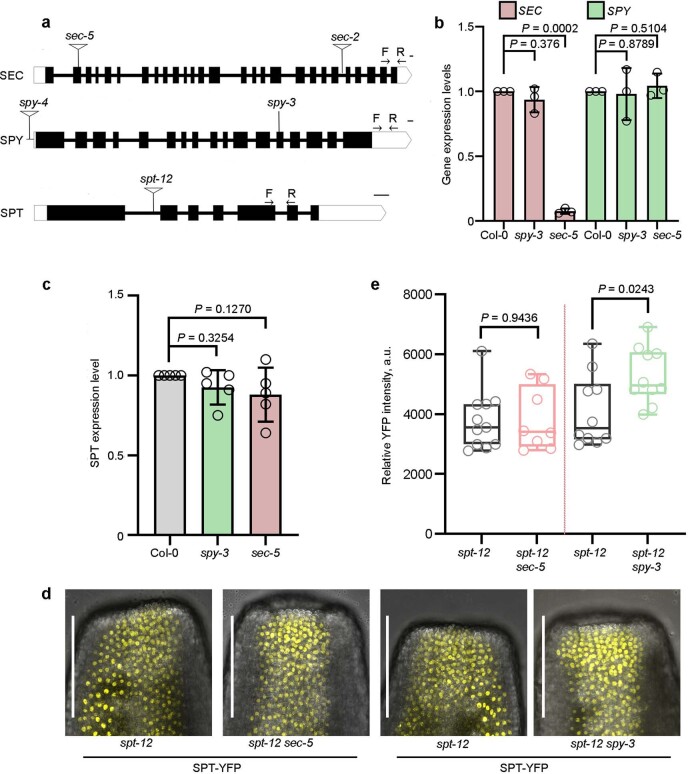
Quantification of *SPT*, *SPY*, and *SEC* expression patterns. **a**, Illustration of the SEC, SPY and SPT gene structure indicating the T-DNA (*sec-5, sec-2, spy-4*, *spt-*12) and point-mutation (*spy-3*) lines and qRT-PCR primers (arrows F and R) used for these experiments. Scale bars represent 100 bp. **b**, Quantification of *SEC* and *SPY* expression by qRT-PCR in mutant inflorescences of the transferase enzymes *sec-5* and *spy-3*. Values shown are means ± SD from three biological repeats. *P* values indicate two-tailed *t* test results. **c**, Quantification of *SPT* expression by qRT-PCR in mutant inflorescences of the transferase enzymes *sec-5* and *spy-3*. Values shown are means ± SD from three biological repeats. *P* values indicate two-tailed *t* test results. **d**, z-stack confocal images of stage-10 gynoecia of SPT complementation line in *sec-5* and *spy-3* backgrounds compared to their respective segregating control, *spt-12/pSPT:SPT:sYFP*. Scale bars represent 100 μm. **e**, Quantification of the YFP signal intensity in nuclei of gynoecia of the genotypes depicted in panel (d). The box plots indicate the median (line within the box), the lower and upper quartiles (box), and the largest and smallest data points (whiskers). All data points are plotted in the graph. For *spt-12/pSPT:SPT:sYFP* in *sec-5* background, n = 11 for control (*spt-12*) and n = 8 for *spt-12 sec-5*, respectively; For *spt-12/SPT::SPT-sYFP* in *spy-3* background, n = 10 for both control (*spt-12*) and *spt-12 spy-3*. The *P* values indicate results of two-tailed *t* test. Source data

To assess whether modifications by *O*-GlcNAc and *O*-fucose on SPT were dependent on SEC and SPY activity, respectively, we crossed the *spt-12/SPT*::*SPT–sYFP* complementation line with a single loss-of-function mutant of SEC (*sec-5*)^33^ and a catalytically redundant mutant of SPY (*spy-3*)^34^ (Extended Data Fig. 2a). Using *spt-12;sec-5/SPT*::*SPT–sYFP* and *spt-12;spy-3/SPT*::*SPT*–*sYFP* inflorescences, we performed a similar HCD MS/MS analysis and compared the percentage of modifications in relation to the *spt-12/SPT*::*SPT–sYFP* segregating controls. Since *O*-fucosylation was hardly detected on peptide_2, we focused this analysis on peptide_1. Our data showed that *O*-fucose modification was completely abolished on peptide_1 in the *spy-3* background, while *O*-GlcNAc was present at comparable levels (Fig. 1e). In contrast, in the *spt-12;sec-5/SPT*::*SPT–sYFP* line, we did not observe any change in the percentage of detectable *O*-fucose, while *O*-GlcNAc was no longer detected in this background (Fig. 1f). This analysis demonstrates that SPY and SEC modify SPT by *O*-fucose and *O*-GlcNAc, respectively, targeting both shared and specific residues. Our analysis highlights that the enzymes do not compensate for each other’s activity in their respective mutant backgrounds, since we did not observe changes in transcript levels of *SPY* and *SEC* in *sec-5* and *spy-3* inflorescences, respectively (Extended Data Fig. 2b), or an increase in their enzymatic activity as assessed by PTMs recovered on SPT (Fig. 1e,f).

**Extended Data Fig. 3:**
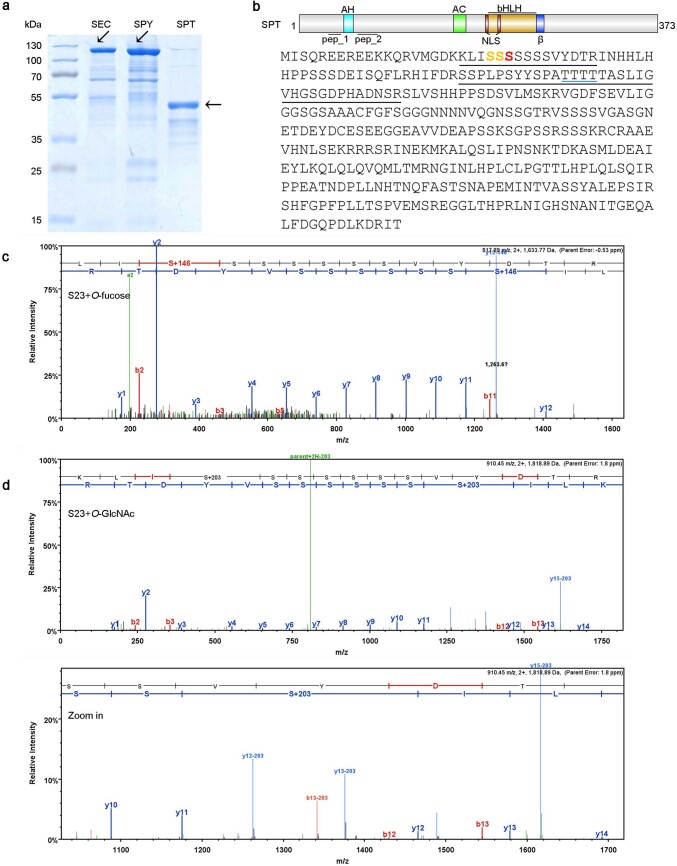
SPT is modified by *O*-GlcNAc and *O*-fucose via SEC and SPY *in vitro*. **a**, SDS-PAGE gel showing purified SEC, SPY and SPT recombinant proteins from *E. coli*. The arrows indicate the band of the proteins at the expected size, *that is*, 10xHis-MBP-5TPR-SEC (SEC), 112 kDa; 10xHis-MBP-3TPR-SPY (SPY), 107 kDa; and 6xHis-SPT (SPT), 44 kDa. The panel shows representative results obtained from at least three repetitions. **b**, Schematic representation of SPT protein domains and amino acid sequence (pep_1 and pep_2 are underlined in black) showing a summary of the residues found modified *in vitro* by *O-*fucose (red residue) and both modifications (*O-*GlcNAc and *O*-fucose, yellow residues) on pep_1, from HCD-MS experiments. The spectra indicate that modification by *O-*GlcNAc can occur on pep_2 around residues T71-T74 (blue underline). **c**, **d**, Representative HCD spectra indicating that SPT pep_1 is modified by *O-*fucose (c) and *O-*GlcNAc (d) (zoom-in spectrum, lower panel) on residue Ser23.

To confirm the direct PTM of SPT by *O*-GlcNAc and *O*-fucose through SEC and SPY, we performed in vitro enzymatic assays. In these assays, we tested the ability of recombinant SEC (5TPR–SEC)^26^ and SPY (3TPR–SPY)^26^ to directly modify the full-length SPT protein (6xHis–SPT) (Extended Data Fig. 3a) in the presence and absence of their specific donor substrate (UDP–GlcNAc and GDP–fucose). Collectively, we found that the HCD spectra obtained in the in vitro experiments recapitulated the in vivo findings, showing that *O*-GlcNAc and *O*-fucose can modify Ser23 to Ser25 of SPT (Extended Data Fig. 3b–d).

Furthermore, no differences were observed for *SPT* transcript levels from *spy-3* and *sec-5* inflorescences compared to the wild type (Extended Data Fig. 2c), and no reduction in SPT stability (as measured by the intensity of the SPT–YFP signal) or alterations in its subcellular localization were observed in the single *spy-3* and *sec-5* mutants (Extended Data Fig. 2d,e). Altogether, our findings corroborate the idea that SEC and SPY work upstream of SPT, acting at the post-translational level.

Our data thus show that specific residues of SPT can host both *O*-GlcNAc and *O*-fucosyl moieties, which are attached by SEC and SPY in vivo and in vitro. Moreover, our data suggest that SEC and SPY may work redundantly but do not compensate for one another in the inflorescences of *Arabidopsis*.

### SPT interacts in vivo and in vitro with SEC and SPY

**Fig. 2:**
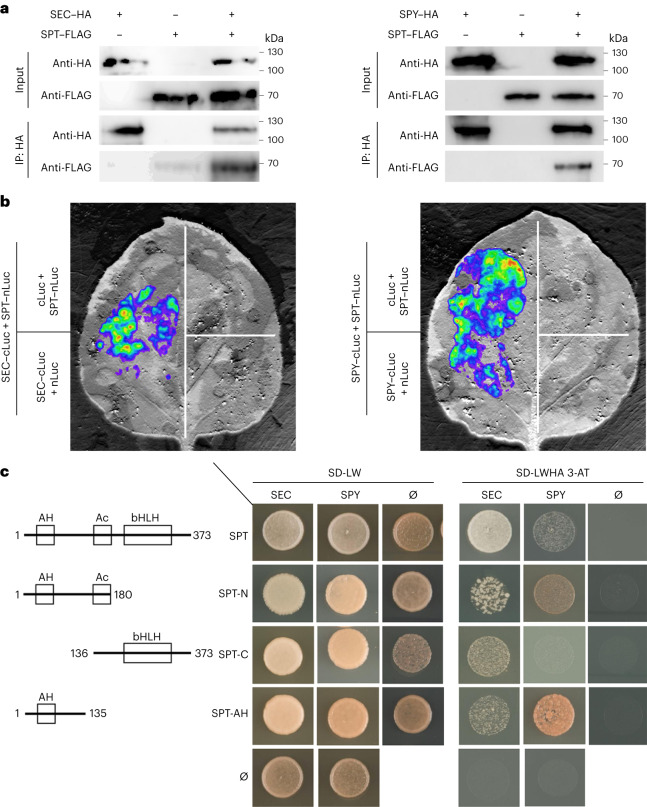
SPT directly interacts with SEC and SPY. **a**, Co-IP experiments performed in tobacco leaves showing the full-length SPT–FLAG protein co-immunoprecipitated with full-length SEC–HA (left) or SPY–HA (right) recombinant proteins. The panel shows representative results from one of three independent experiments. **b**, Split-luciferase assay performed in tobacco leaves showing the interaction between full-length SPT–nLuc with both SEC–cLuc (left) and SPY–cLuc (right). The panel shows representative results from one of three independent experiments. **c**, Y2H assay displaying SPT domains (left) sufficient for interactions with full-length SPY and SEC proteins. Positive co-transformants were selected on SD-LW media (middle) and grown on SD-LWHA, 2.5 mM 3-AT media (right) to determine strong interactions. Ø, empty vector. Source data

**Extended Data Fig. 4:**
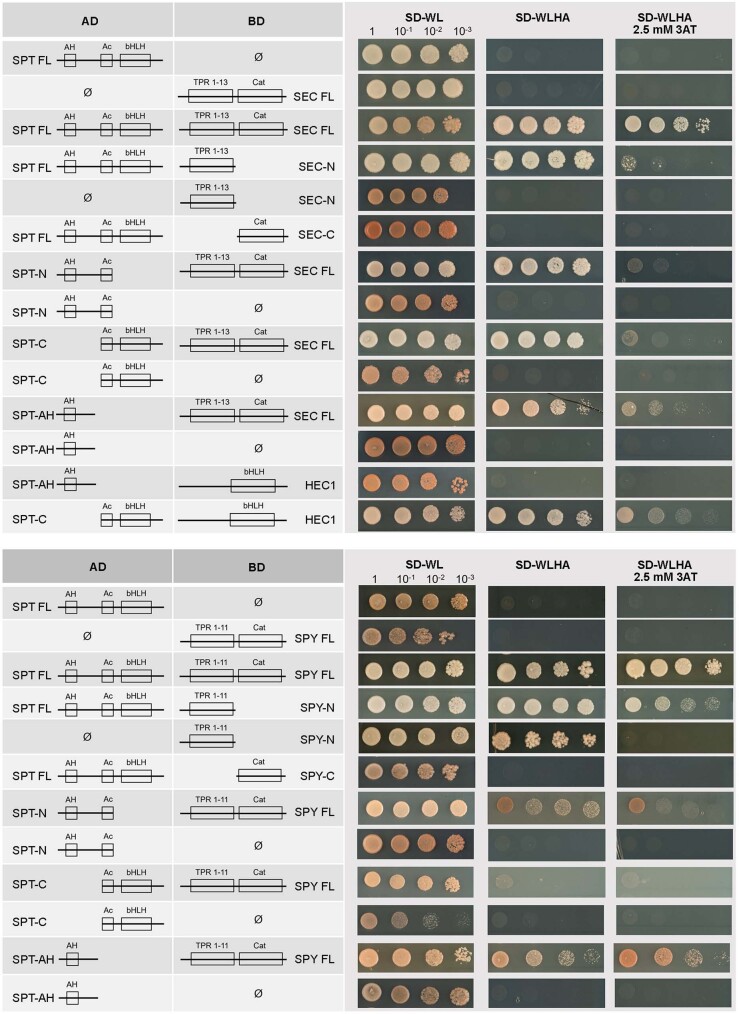
SEC and SPY interact with SPT via their N-terminal domains. Yeast-two-Hybrid experiments to determine SPT-SEC (top panel) and SPT-SPY (bottom panel) protein-protein interactions. Full-length (FL) proteins and functional domains (TPRs and Cat) structures are depicted on the left panel. HEC1 (top panel) was used as negative and positive control to test interactions with SPT-AH domain and the SPT-C (Ac plus bHLH domains combined), respectively. The assays were performed using SEC and SPY as the bait (BD) and SPT as prey (AD). Growth on selection media SD-LWHA supplemented with 2.5 mM 3-AT indicates strong interactors. The dilutions examined in the experiments encompass ratios of 1:1, 1:10, 1:100, and 1:1000.

To support a role for SEC and SPY in controlling SPT at the post-translational level, we tested whether these enzymes could directly interact with SPT. To this end, we employed co-immunoprecipitation (Co-IP) and split-luciferase complementation assays in *Nicotiana benthamiana* leaves (Fig. 2a,b) alongside yeast two-hybrid (Y2H) experiments (Fig. 2c and Extended Data Fig. 4), which all demonstrated direct interactions between full-length SPT–SPY and SPT–SEC proteins. Moreover, Y2H experiments also showed that both SPY and SEC bind to SPT via their N-terminal tetratricopeptide repeats (TPRs)^26^, TPRs 1–11 of SPY and TPRs 1–13 of SEC, while the carboxy-terminal catalytic domain showed no growth of the yeast cells on selective media (Extended Data Fig. 4).

Most of the residues modified on SPT were positioned at peptides flanking an amphipathic helix (AH) at the N terminus of SPT (Fig. 1a), a domain that supports the transcriptional activation activity of SPT^35^. Thus, to dissect the domains responsible for mediating the enzyme interactions with SPT, we performed Y2H experiments using the full-length sequences of SPY and SEC in combination with truncated versions of SPT. Our results showed the SPT N-terminal domain (spanning residues 1–180) containing the AH and acid (Ac) domains^35^, as well as a fragment containing only the AH domain (residues 1–135), were both sufficient to produce positive interactions with full-length SEC and SPY proteins (Fig. 2c and Extended Data Fig. 4) but did not interact with HEC1—a known interactor of SPT (Extended Data Fig. 4). In contrast, the SPT C-terminal truncation (residues 136–373), including both the Ac and bHLH domains, did not interact with SPY but could interact with SEC as well as HEC1 (Fig. 2c and Extended Data Fig. 4). These results are in line with a working model in which the binding of SPT with SPY occurs through the N-terminal domain of SPT containing the modified residues flanking the AH domain, while SEC interacts with the full-length SPT protein. These findings also provide insights into the signalling modulation at the N terminus of SPT, a crucial molecular site for both transcriptional regulation and carpel patterning^35^.

### SEC and SPY synergistically control style morphogenesis

**Fig. 3:**
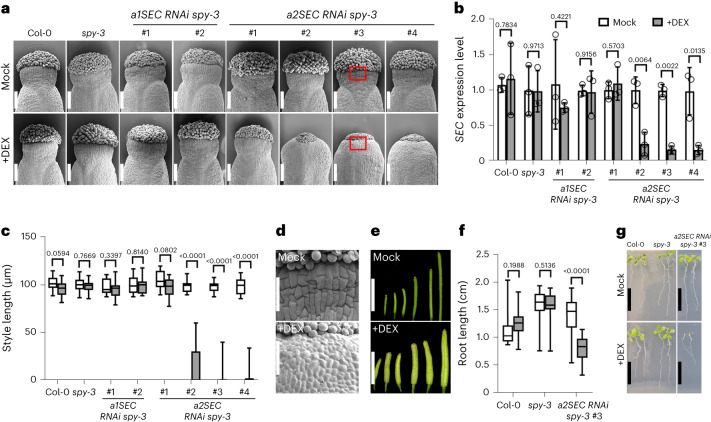
Loss of SEC function in a *spy* mutant background affects both vegetative and reproductive development. **a**, SEM analysis of stage-12 gynoecia of Col-0, *spy-3* and *SEC* *RNAi* *spy-3* transgenic lines (two lines of *a1SEC RNAi spy-3* and four lines of *a2SEC RNAi spy-3*) treated with either mock (top) or DEX (bottom). Scale bars, 100 μm. **b**, qRT-PCR analysis of *SEC* expression levels from inflorescences of *SEC* *RNAi* *spy-3* transgenic lines (*a1SEC*s and *a2SEC*s) and controls, treated with either mock (white bars) or DEX (grey bars). The values shown are means ± s.d. from three biological repeats. The *P* values indicate two-tailed *t*-test results. **c**, Measurement of style length of the genotypes depicted in **a**,**b**. The box plots indicate the median (the line within the box), the lower and upper quartiles (the box edges), and the largest and smallest data points (the whiskers). For Col-0, *spy-3* and *a1SEC RNAi spy-3* transgenic lines, *n* = 15 for mock and DEX treatments; for the *a2SEC* *RNAi* *spy-3* transgenic lines, *n* = 15 for mock and *n* = 18 for DEX treatment. Note that the strong reduction in style elongation (**a**,**c**) correlates with low levels of *SEC* expression (**b**). The *P* values are indicated in the graph following two-tailed Mann–Whiney tests. **d**, SEM micrographs showing wild-type and *SEC* *RNAi* *spy-3* (*a2SEC* line 3) styles (see the red boxes in **a**) displaying defects in cell elongation. Scale bars, 50 μm. The panel shows representative results from one of three independent experiments. **e**, Representative images of fruits in different stages of development after mock (top) and DEX (bottom) treatments of wild-type and *SEC* *RNAi* *spy-3* (*a2SEC* line 3) inflorescences. Scale bars, 1 cm. **f**, Root length measurements of seven-day-old Col-0, *spy-3* and *SEC* *RNAi* *spy-3* (*a2SEC* line 3) seedlings grown on either mock or DEX plates. Significant differences are indicated in the graph following two-tailed Student’s *t*-tests. The box plots indicate the median (the line within the box), the lower and upper quartiles (the box edges), and the largest and smallest data points (the whiskers). *n* = 17 seedlings were analysed for each genotype and each treatment. The *P* values indicate the results of two-tailed *t*-tests. **g**, Representative images of 13-day-old Col-0, *spy-3* and *SEC* *RNAi* *spy-3* (*a2SEC* line 3) seedlings grown on either mock or DEX plates. Scale bars, 1 cm. Source data

**Fig. 4:**
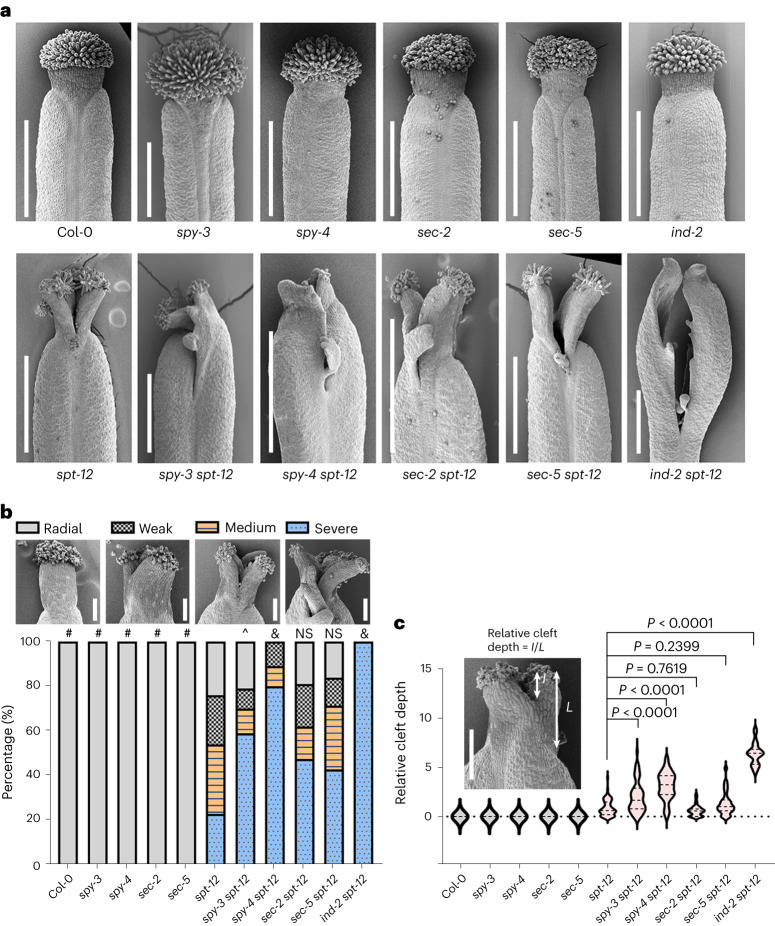
The symmetry-breaking phenotype of the *spt-12* mutant style is epistatic to both *sec* and *spy* mutations. **a**, Representative SEM micrographs of stage-12 gynoecia of single and double mutants. Scale bars, 500 μm. The experiment was performed at least three times for each genotype. **b**, Quantification of the frequency and severity of the bilaterally symmetric defective styles observed in the double *sec* *spt* and *spy* *spt* mutants compared with their parental lines. Phenotypes were grouped in the following four categories: radial (wild-type-like style, radially symmetric), weak (shallow cleft at the organ’s distal tip), medium (cleft running through the middle of the style) and severe (deep cleft spanning the style into the ovary region). Fifty gynoecia were analysed for each genotype. The phenotypic classes were compared with *spt-12* using 4 × 2 contingency tables followed by Pearson’s *χ*^2^ test. The two-tailed *P* values are as follows: &, *P* < 0.0001; #, *P* < 0.00001; ^ *P* = 0.000019; NS, not significant *P* > 0.05 (*sec-2 spt-12* versus *spt-12*, *P* = 0.0249; *sec-5 spt-12* versus *spt-12*, *P* = 0.1007). *P* values <0.01 were considered statistically significant. Scale bars, 100 μm. **c**, Quantification of the severity of the bilateral split style (relative cleft depth) was measured as the depth of the cleft (*I*) divided by the style length (*L*). More than 60 gynoecia were analysed for each genotype. The internal dashed lines indicate the median and quartiles. Phenotypic classes were compared with *spt-12* using ordinary one-way analysis of variance (ANOVA) multiple comparisons. Scale bar, 100 μm. Source data

If the activity of *Arabidopsis* SEC and SPY controls style development at the gynoecium apex, defects in style specification or elongation or a break in radial symmetry (similar to *spt*) should be observed in mutants for *sec* and *spy*. Despite decades of genetic analysis on single mutants for these two enzymes, no defects have ever been reported^30,36,37^. Accordingly, our scanning electron microscopy (SEM) analysis of loss-of-function *spy-4* (ref. ^37^) and *sec-5* single mutant gynoecia, as well as the catalytically redundant mutants *spy-3* and *sec-2* (ref. ^36^), did not show any significant defects in style formation (Figs. 3a and 4a). The correct style development observed in *sec* and *spy* mutants is in line with normal *SPT* expression within the style of these single mutants (Extended Data Fig. 2c,d). This may suggest a redundant role for both enzymes in controlling the morphogenesis of the gynoecium apex.

**Extended Data Fig. 5:**
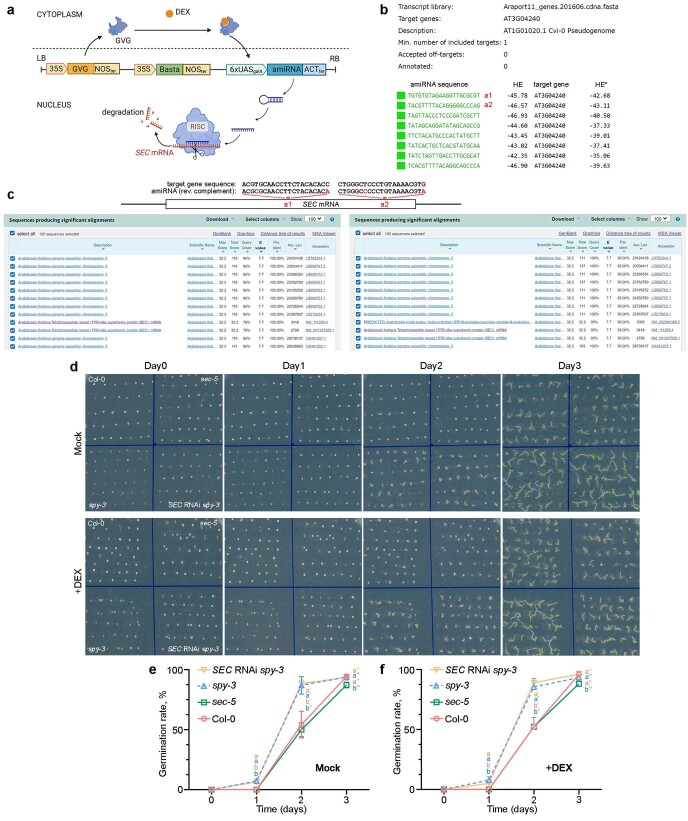
DEX-inducible *SEC* RNAi cloning strategy and seed germination rate analysis. **a**, Schematic illustration of the working principle of the construct for DEX inducible RNAi of *SEC*. **b**, Prediction of highly specific amiRNA sequences for *SEC* gene (AT3G04240) in WMD3 (http://wmd3.weigelworld.org/cgi-bin/webapp.cgi). The top-ranking two sequences (a1 and a2, in red) were selected for analysis. HE and HE* indicate the hybridization energy of the amiRNA to a perfectly matching complement (HE, column 2) and to the target site in the target gene (HE*, column 4); identifier of the target gene (column 3). **c**, Schematic illustration of the a1 and a2 complementary sequences on the *SEC* mRNA (top), highlighting the mismatched nucleotides on the amiRNA guides, in red. Bottom, BLAST results for the *a1SEC* (bottom left) and *a2SEC* (bottom right) guide specificity in the NCBI database. The BLAST results show very high specificity for both sequences in Arabidopsis. **d**, Representative images of Col, *spy-3*, *sec-5* and *SEC RNAi spy-3* (*a2SEC* line 3) seeds germinated on either mock or DEX plates. **e,f**, Statistical analysis of the germination rate of Col, *spy-3*, *sec-5* and *SEC RNAi spy-3* (*a2SEC* line 3) seeds germinated on either mock (e) or DEX (f) plates. More than 300 seeds from four biological repeats for each genotype were analysed. Values shown are means ± SD. Different letters at the same timepoint indicate significant differences (*P* < 0.05) determined by ordinary one-way ANOVA (Tukey’s multiple comparison test). The exact *P* values are provided in the Statistical Source Data1 file. The colours of the letters are consistent with the genotype in the graph. Source data

Varying effects on carpel development were unveiled through haplo-deficiency genetic analysis of the SEC and SPY functions—that is, four-carpel ovaries and pin-like flowers^36^. To overcome the embryo-lethal effect elicited by the elimination of both enzymatic activities^30,36^ and reveal the synergistic post-embryonic roles of the two enzymes (encompassing style development), we produced a dexamethasone (DEX)-inducible *SEC* *RNAi* construct to lower *SEC* mRNA levels in the *spy-3* background (*SEC RNAi spy-3*) (Extended Data Fig. 5a). Two specific target guides were designed (named *a1SEC* and *a2SEC*) for SEC, ensuring low off-target scores (Extended Data Fig. 5b,c). Only the *Arabidopsis* transgenic lines generated using the *a2SEC* guide showed reduced levels of *SEC* transcripts, as determined by quantitative real-time PCR (qRT-PCR) experiments conducted from DEX-sprayed inflorescences (Fig. 3b). Three independent transgenic lines recovered using the *a2SEC* guide were subsequently phenotypically analysed and compared to Col-0, *spy-3* and *SEC RNAi spy-3* lines that did not exhibit *SEC* downregulation, as negative controls (Fig. 3a,b). We observed defects in style morphogenesis after DEX treatment of transgenic lines with reduced levels of *SEC* expression—that is, a strong reduction in style length (Fig. 3a,c), a lack of wax/crenulations of stylar cells (used as a differentiation marker for style) (Fig. 3d), a reduction of cell elongation below the apical stigmatic tissue (Fig. 3d) and a consistent reduction in fruit length (Fig. 3e). The short-style phenotype and compromised cell elongation observed in the DEX-induced *SEC RNAi spy-3* is remarkably different from the split-style phenotype of *spt* (Fig. 4a). It is consistent with a lack of apical-basal anisotropic growth displayed by cells at the developing apical gynoecium, where the style forms^38,39^. These results thus reveal a previously unrecognized role for SEC and SPY in style development and suggest that SEC and SPY control early developmental processes of style morphogenesis, presumably via a redundant and synergistic control of style- and fruit-specific targets. Consistent with a positive role for SEC and SPY in post-embryonic plant organogenesis, we observed a reduction of root length in *SEC RNAi spy-3* (*a2SEC*) seedlings grown on DEX media for seven days compared with mock-treated samples and DEX-treated Col-0 and *spy-3* parental line (Fig. 3f). Moreover, we observed arrest of growth after 13 days of growth on DEX media of the *SEC RNAi spy-3* genetic background (Fig. 3g). Interestingly, while knocking down SEC activity did not affect the germination rate differently from the *spy-3* control (Extended Data Fig. 5d–f), the reduction of root growth and lethality observed at the seedling stage confirm the fundamental roles of both enzymes in post-embryonic plant development.

Next, to investigate the role of SEC and SPY in style radialization via regulation of SPT activity, we analysed the gynoecia of *spt* *sec* (*spt-12* *sec-5* and *spt-12* *sec-2*) and *spt* *spy* (*spt-12* *spy-4* and *spt-12* *spy-3*) double mutants (Fig. 4a). Our genetic analysis showed that *spt* *sec* double mutants displayed a similar phenotype to the segregating *spt-12* control, while *spt* *spy* double mutant combinations increased the frequency and severity of the *spt* split-style phenotype by strongly augmenting the percentage of bilateral versus radial styles observed (Fig. 4b), as well as the depth of the medial cleft (measured as the ratio between the cleft length and the style length) (Fig. 4c). These data indicate that *spt* is epistatic to the loss of both SEC and SPY enzymatic activities and suggest that *O*-fucosylation of other key regulators and cellular processes of style development may be regulated in parallel by SPY (for example, apical-basal anisotropic growth)^38,39^.

**Extended Data Fig. 6:**
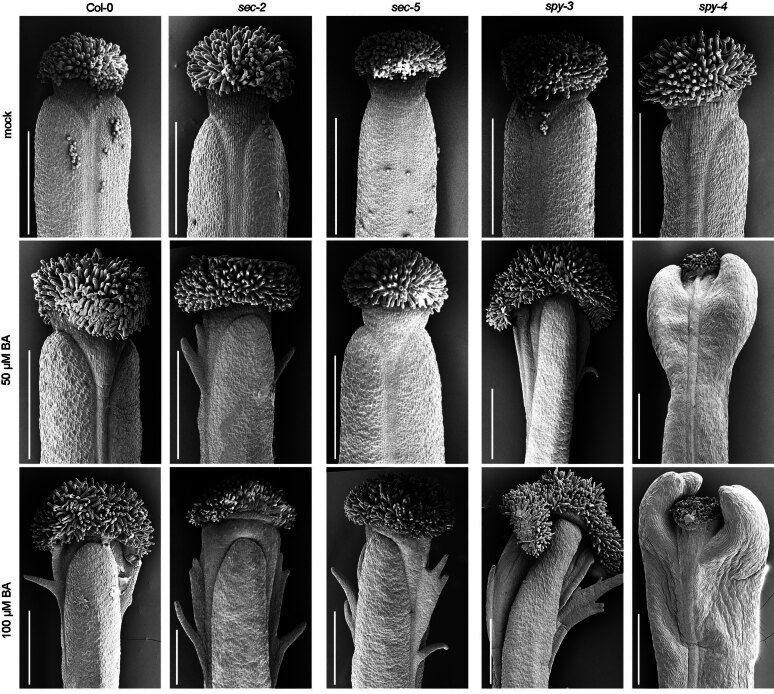
*spy* mutant gynoecia are hypersensitive to cytokinin treatments. Representative SEM images of stage-13 gynoecia of Col-0, *sec-2*, *sec-5*, *spy-3* and *spy-4* genetic backgrounds after pharmacological treatment with cytokinin (either 50 μM or 100 μM BA) and mock. Scale bars represent 500 μm. The panels show representative results from one of three independent experiments.

To further examine this possibility, *sec* and *spy* mutant gynoecia were treated with CK. No defects were displayed by *sec* mutants, but for the *spy* mutants we observed extensive proliferation of the medial-apical region that connects the stigma and replum (*spy-3*) as well as ectopic, unbalanced growth of the lateral shoulders (*spy-4*) (Extended Data Fig. 6), a phenotype associated with CK hypersensitivity that has not been previously observed. These data confirm that SPY plays multiple roles during gynoecium development. They also corroborate the idea that SPY and SPT work in similar pathways, as they both repress the CK proliferating output at the gynoecium apex, which is relevant as it provides a genetic framework for an antagonistic SPY–CK interaction.

### Specific Ser/Thr residues of SPT promote style radialization

**Fig. 5:**
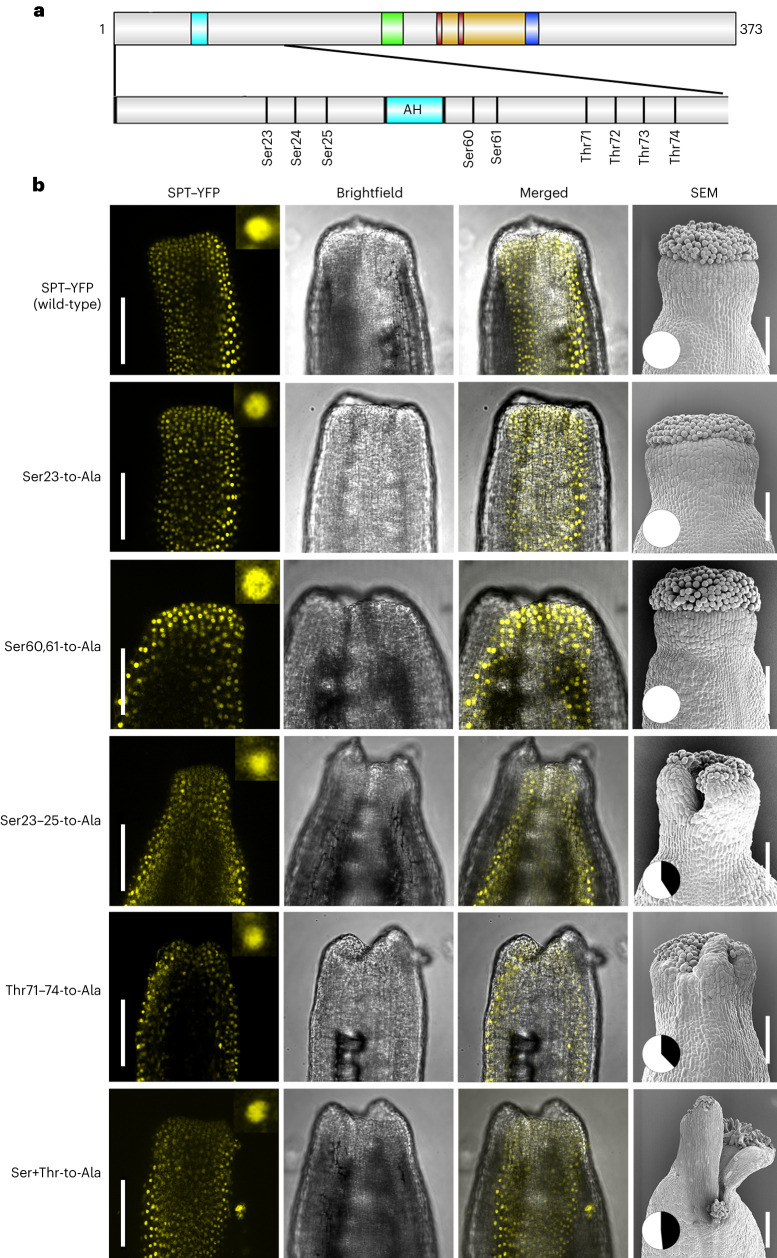
Genetic complementation analysis of SPT revealed a positive role for *O-*glycosyl-modified residues in *Arabidopsis* radial style development. **a**, Schematic representation of SPT residues modified by *O*-fucose and *O*-GlcNAc used for point mutations: the Ser and Thr residues depicted in the panel were mutated to Ala. Possible modifications of Ser23, Ser24 and Ser25 were confirmed both in vivo and in vitro. Modifications of Thr71–Thr74 were indicated by the EThcD results and were previously identified^21^. Modifications of Ser60 and Ser61 were used as a negative control. **b**, Representative confocal images of gynoecia at stages 10–11 and SEM images (right) of gynoecia at stage 12 of the *SPT–YFP* complementation and point-mutation lines. YFP signal is detected at the style and in the nuclei (inset: close-up of a nucleus with SPT–YFP signal from gynoecia). The pie charts in the SEM pictures represent the percentage of radial (white) versus bilateral (black) styles observed in each genetic background. Three independent lines for each construct were analysed, yielding similar results. Scale bars, 100 μm. Source data

**Extended Data Fig. 7:**
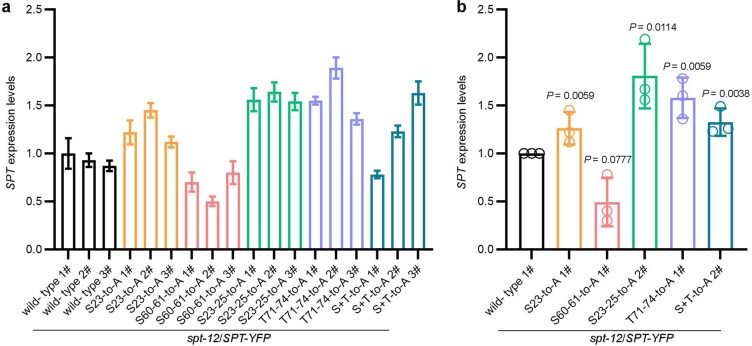
Molecular characterization of *spt-12*/*SPT-YFP S/T-to-A* point mutation lines. **a**, Comparison of *SPT* transcript levels in young inflorescences of three independent *spt-12/SPT-YFP* complementation (wild-type sequence) and point mutation lines, by qRT-PCR experiments. The experiment was performed once on three independent transgenic lines per construct, with four technical repeats. Values shown are means ± SEM. **b**, Quantification of *SPT* transcript levels in selected transgenic lines of *spt-12/SPT-YFP* complementation and point mutation lines depicted in the graph. Values shown are means ± SD from three biological repeats. The *P* values indicated comparison with wild-type by two-tailed student’s *t* test. Source data

To test the effect of *O*-glycosyl decorations on SPT function during radial symmetry establishment at the gynoecium apex, we carried out a detailed mutant complementation assay by producing a series of loss-of-*O*-glycosylation mutant variants of SPT (expressed under the *SPT* native 5-kb promoter^40^) and analysed their ability to complement the *spt* split-style phenotype. To this end, specific Ser and Thr residues targeted by SEC and SPY in vivo and in vitro on SPT peptide_1 (Fig. 1a and Extended Data Figs. 1 and 3) and peptide_2 (ref. ^21^) were mutated to Ala to mimic the loss of modification^41^. We predicted that the lack of style complementation would indicate a positive functional role for those residues and the associated PTMs in sustaining SPT function. We produced and analysed the following mutant versions of SPT: Ser23-to-Ala; Ser23, Ser24 and Ser25-to-Ala (hereafter Ser23–25-to-Ala, on peptide_1); Thr71, Thr72, Thr73 and Thr74-to-Ala (hereafter Thr71–74-to-Ala^21^, on peptide_2); Ser23, Ser24, Ser25, Thr71, Thr72, Thr73 and Thr74-to-Ala (hereafter Ser+Thr-to-Ala, on peptide_1 and peptide_2); and Ser60 and Ser61-to-Ala (Ser60,61-to-Ala, as a negative control, on peptide_2) (Fig. 5a). First, to exclude the possibility that changes in the amino acid sequence of SPT would lead to alterations in transcript levels and protein stability or localization, we fused the SPT wild-type and mutant sequences with yellow fluorescent protein (YFP) and carried out qRT-PCR experiments (Extended Data Fig. 7a,b) and confocal microscopy analysis (Fig. 5b). None of the lines examined showed a significant reduction in *SPT* mRNA levels (Extended Data Fig. 7a,b). Furthermore, confocal microscopy analysis showed that a clear YFP signal was present at the apex of gynoecia—where style and stigmatic tissue form—expressing the wild-type SPT complementation line as well as the Ser23-to-Ala (peptide_1) and Ser60,61-to-Ala (peptide_2) point mutations (Fig. 5b). Accordingly, SEM analysis showed that the *spt* phenotype was complemented by Ser23-to-Ala and Ser60,61-to-Ala mutations to a similar level as the wild-type sequence, equal to 100% of radial styles (Fig. 5b). In contrast, despite clear SPT nuclear expression at the gynoecium apex of Ser23–25-to-Ala (peptide_1), Thr71–74-to-Ala (peptide_2) and Ser+Thr-to-Ala (both peptides), these point-mutation lines displayed a high percentage of bilateral styles, resembling the *spt* mutant. Specifically, 40.9%, 37.7% and 48.4% of the styles observed in the aforementioned transgenic lines were unfused—that is, they had the split-style phenotype (Fig. 5b).

These results thus indicate a functional role for specific SPT residues in style radial symmetry development in *Arabidopsis* via a mechanism distinct from transcriptional expression, protein stability and cellular localization.

### *O*-glycosyl PTMs on SPT promote its transcriptional activity

Since SPT binds DNA (and thus regulates transcription) as a dimer^4^, we hypothesized that SEC and SPY may impact its ability to interact with protein partners and/or specific promoters. To understand how PTMs of SPT may play a role in orchestrating style morphogenesis, we tested whether SEC and SPY could enhance the formation of the SPT–SPT homodimers and/or SPT–IND and SPT–HEC1 heterodimers essential for correct style morphogenesis.

**Fig. 6:**
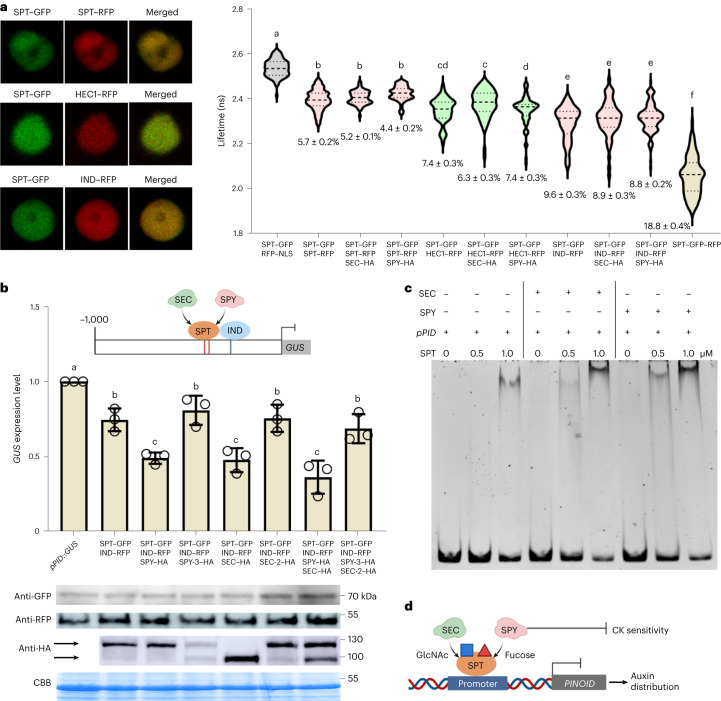
SEC and SPY promote the SPT-mediated repression of *PID* transcription rather than its ability to form homo- and heterodimers. **a**, Left, FRET–FLIM assay showing that both SEC and SPY have no effect on the strength of the interaction between SPT–GFP and its interacting partners SPT–RFP, IND–RFP and HEC1–RFP, in nuclei of co-infiltrated tobacco leaves. The experiments were repeated at least three times for each combination; more than 60 nuclei were used for quantification for each combination. Right, the FRET efficiency (%) is indicated in the violin plots. The internal dashed and dotted lines indicate the median and quartiles. Different letters indicate significant differences (*P* < 0.05) tested by ordinary one-way ANOVA multiple comparisons. The exact *P* values are provided in the Source data. **b**, Top, schematic representation of the 1-kb promoter of *PID* used in the transactivation assays, including the TF binding sites (G-boxes, indicated by the red lines, and E-box, indicated by the blue line). Middle, quantification of *GUS* expression by qRT-PCR experiments showing that both SEC–HA and SPY–HA enhance the transcriptional downregulation triggered by SPT and IND. The optical density (OD) values employed were OD = 0.1 for *SPT–GFP* and *IND–RFP* and OD = 0.5 for *SEC–HA* and *SPY–HA*, in each experiment. The experiments were repeated three times for each combination (*n* = 3). The values shown are means ± s.d. Different letters indicate significant differences (*P* < 0.01) tested by ordinary one-way ANOVA multiple comparisons. The exact *P* values are provided in the Source data. Bottom, immunodetection of SPT–GFP, IND–RFP, SEC–HA and SPY–HA from *N. benthamiana* *Agrobacterium*-infiltrated leaves used for the transactivation assays. The Coomassie brilliant blue (CBB) bands were used as a sample loading control. **c**, EMSA experiments showing that SEC (5TPR–SEC) and SPY (3TPR–SPY) both enhance the binding of SPT (6xHis–SPT) to the region of the *PID* promoter (171-bp *pPID*). Similar results were obtained from two independent experiments. **d**, Schematic working model showing that SEC and SPY act upstream of SPT to attach *O-*GlcNAc (blue square) and *O-*fucose (red triangle), which in turn promotes the binding of SPT to the *PID* promoter and/or its transcriptional activity. In this way, SEC and SPY contribute to the fine-tuning of auxin distribution while repressing CK sensitivity at the gynoecium apex, ultimately promoting style morphogenesis. Source data

To test whether SEC and SPY could impact SPT dimerization events, we performed fluorescence resonance energy transfer (FRET)–fluorescence-lifetime imaging microscopy (FLIM) quantitative assays in tobacco leaves to assess whether the co-expression of *Agrobacterium* harbouring either SEC–HA (*35S*::*SEC–3xHA*) or SPY–HA (*35S*::*SPY–3xHA*) would lead to a further reduction in the lifetime of the FRET donor (GFP) compared with the formation of the homodimer SPT–GFP;SPT–RFP (*35S*::*SPT*::*GFP*;*35S*::*SPT–RFP*) and the heterodimers SPT–GFP;IND–RFP *(35S*::*SPT*::*GFP*;*35S*::*IND–RFP*) and SPT–GFP;HEC1–RFP (*35S*::*SPT*::*GFP*;*35S*::*HEC1–RFP*) (Fig. 6a).

**Extended Data Fig. 8:**
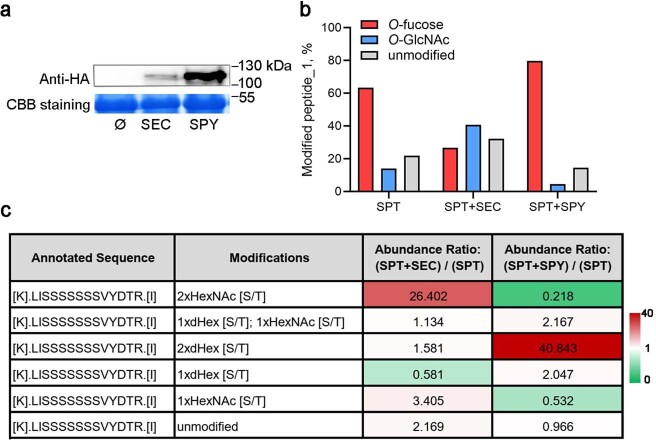
SPT is modified by *O*-GlcNAc and *O*-fucose via SEC and SPY in tobacco leaves. **a**, Immunodetection of SEC-HA (*35S::SEC-3xHA*) and SPY-HA (*35S::SPY-3xHA*) from Agro-infiltrated *N. benthamiana* leaves. The CBB (Coomassie Brilliant Blue) bands were used as sample loading control. The data shows representative results from one of three independent experiments. **b**, Percentage of *O*-GlcNAc-modified, *O*-fucose-modified or unmodified SPT peptide_1 detected in HCD MS/MS experiments from tobacco leaves infiltrated with GV3101 strains carrying either construct of *SPT* alone or with *SEC* and *SPY*. The graph shows representative data generated from one of the three independent biological repeats. **c**, Table depicting the abundance ratio of SPT peptide_1 modified by *O*-GlcNAc (HexNac) and *O*-fucose (dHex), resulting from co-expression with SEC and SPY, in comparison to the abundance of the same peptide observed in SPT-alone expression. The colour scale indicates the range of ratio values. Source data

To begin, we confirmed the expression of SEC–HA and SPY–HA recombinant proteins by western blotting (Extended Data Fig. 8a). We then tested whether SEC and SPY could modify SPT in tobacco leaves, by means of HCD MS/MS experiments (Extended Data Fig. 8b,c). In line with our in vivo and in vitro findings (Fig. 1 and Extended Data Figs. 1c–e and 3b–d), co-infiltration of SEC or SPY with SPT resulted in an elevated percentage of *O*-GlcNAc or *O*-fucose modifications, respectively, detected on peptide_1 (Extended Data Fig. 8b) as well as a higher ratio of modified peptides compared with the SPT control (Extended Data Fig. 8c).

Next, we confirmed a direct interaction in the nuclei co-expressing (;) SPT–GFP;SPT–RFP as well as SPT–GFP;IND–RFP and SPT–GFP;HEC1–RFP (without co-expression of the enzymes) with FRET–FLIM assays. The GFP fluorescence lifetime was significantly shorter in the presence of SPT–GFP;SPT–RFP, SPT–GFP;IND–RFP and SPT–GFP;HEC1–RFP interactions than in the negative control: SPT–GFP;RFP–NLS (which targets the RFP protein in the nucleus) (Fig. 6a). These changes in GFP fluorescence lifetime are consistent with FRET efficiencies of 5.7%, 9.6% and 7.4% for SPT;SPT, SPT;IND and SPT;HEC1, respectively (Fig. 6a). The positive FRET control, SPT–GFP–RFP (in which the two fluorescent tags are both cloned in *cis* to SPT) showed an even stronger decrease in GFP fluorescence lifetime, which translated into a FRET efficiency of 18.8% (Fig. 6a). However, the presence of either SEC–HA or SPY–HA had no significant effect on the FRET efficiencies observed in the presence of interactions between SPT–GFP and its RFP-tagged partners (Fig. 6a). In principle, this would suggest that *O*-GlcNAc and *O*-fucose do not promote the formation of SPT-containing dimers. However, the enzymes may modulate dimer formation via other mechanisms—for example, by conformational rearrangement.

**Extended Data Fig. 9:**
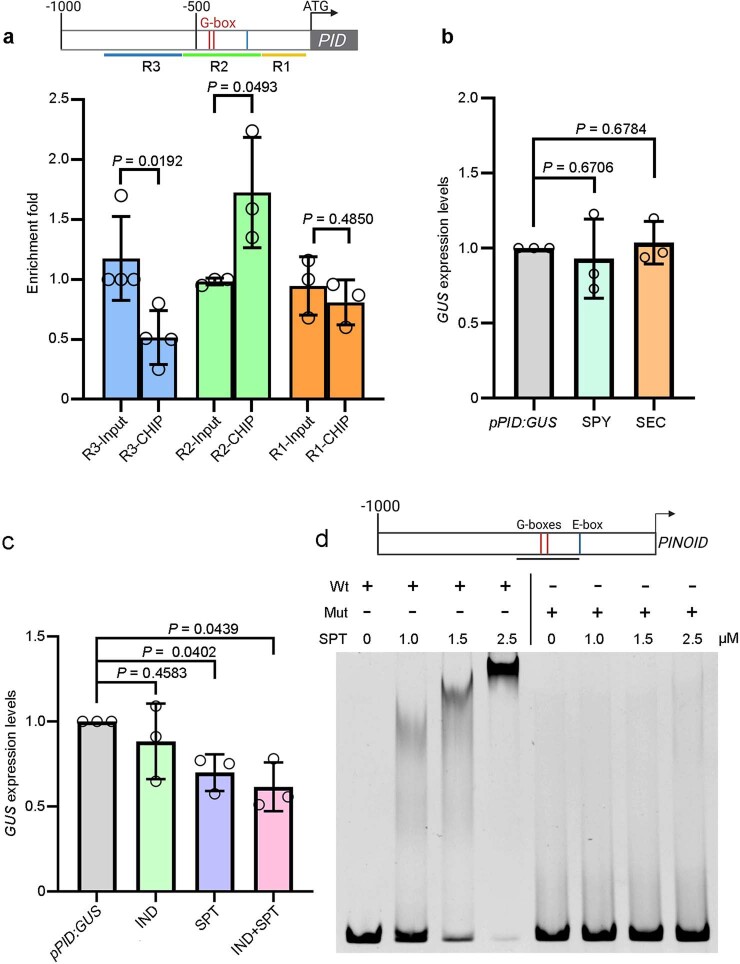
Molecular interaction between SPT and *PINOID* promoter *in vivo* and *in vitro*. **a**, Top, schematic representation of the *PID* promoter fragment including G-boxes (red lines) and E-box (blue line), and the position of the fragments amplified across those *cis-*elements. Bottom, qRT-PCR quantification of ChIP experiments carried out from young inflorescences of *spt-12/pSPT:SPT:sYFP* complementation line. The enrichment of the *SPT* binding to the region (R2) containing the two G-boxes demonstrate SPT binds *in vivo* to the *PID* promoter. Values shown are means ± SD from three biological repeats for R1 and R2; four biological repeats for R3. The *P* values indicate results of two-tailed *t* test. **b**, qRT-PCR quantification of *GUS* expression in transactivation assay from tobacco leaves infiltrated with either the *pPID:GUS* construct alone or with SEC-HA (*35S::SEC-3xHA*) or SPY-HA (*35S::SPY-3xHA*). OD = 0.5 was used for both SEC and SPY in each experiment. Note, SEC and SPY have no effect on p*PID:GUS* expression. Values shown are means ± SD from three biological repeats. The *P* values indicate results of two-tailed *t* test. **c**, qRT-PCR experiments showing quantification of *GUS* expression in transactivation assays from tobacco leaves following infiltration with IND-RFP (IND) and SPT-GFP (SPT) alone or both (IND + SPT) compared to the *pPID:GUS* construct alone. OD = 0.5 was used for both SPT and IND in each experiment. Values shown are means ± SD from three biological repeats. The *P* values indicate results of two-tailed *t* test. **d**, Graphic representation of the *PID* promoter including the 171-bp fragment used in EMSA experiments. Recombinant SPT (6xHis-SPT) binds to wild-type sequences of the G-box (red lines) present in the *PINOID* promoter, in a concentration-dependent manner (left panel). Incubation with a mutated (Mut) G-boxes (from CACGTG to TGATGA) abolish this binding. Similar results were obtained from two independent experiments. Source data

We then investigated whether SEC and SPY would impact SPT-mediated gene expression. The best-characterized downstream target of SPT (and IND) during style development is the *PID* kinase^6,9,42,43^, which is downregulated to support the accumulation of the auxin morphogenic signal within specific medial-apical cells^6^. We thus tested whether the downregulation of *PID* expression by SPT–IND was exacerbated by SEC and SPY by performing a transient transactivation assay in *N. benthamiana* leaves using the same constructs used for the FRET–FLIM experiments and a 1-kb promoter fragment of *PID*, containing the *cis-*elements directly bound by both IND^9,10,42,43^ and SPT in vivo (Extended Data Fig. 9a).

The *PID* promoter was fused to the *GUS* gene as a reporter. To begin, we confirmed that the expression of *SEC–HA* or *SPY–HA* did not affect *GUS* expression (Extended Data Fig. 9b), and the transcriptional read-out of SPT/IND was determined by qRT-PCR experiments using different ODs for co-infiltration (Fig. 6b and Extended Data Fig. 9c). We first tested for equal expression of the co-infiltrated recombinant proteins by western blots and then compared the *GUS* transcription levels (Fig. 6b). The expression of *SPT–GFP* and *IND–RFP* either alone (homodimers) or co-expressed (heterodimer) significantly diminished *pPID*::*GUS* expression levels (Fig. 6b and Extended Data Fig. 9c), while the expression of either *SEC–HA* or *SPY–HA* had no significant effect on transcription alone (Extended Data Fig. 9b). However, co-expression of both *SEC–HA* and *SPY–HA* together with *SPT–GFP* and *IND–RFP* led to a significant further reduction of the background levels of *PID* expression (Fig. 6b). Furthermore, co-expression of the catalytic SPY (*spy-3–HA*) and SEC (*sec-5–HA*) mutant enzymes, together with SPT–GFP and IND–RFP recombinant proteins, led to a reversion of the *GUS* transcriptional levels to the basal repression trigged by the SPT–GFP/IND–RFP heterodimer (Fig. 6b). Altogether, these results demonstrate that both SEC and SPY enhance SPT/IND-mediated repression of *PID* transcription.

To corroborate the idea that *O*-glycosyl PTMs of SPT directly influence its transcriptional activity rather than dimerization events, we tested whether the affinity of SPT for the *PID* promoter was enhanced by SEC and SPY. To this end, we employed electrophoretic mobility shift assay (EMSA) experiments performed using the full-length 6xHis–SPT recombinant protein (Extended Data Fig. 3a), which we showed was able to bind a 171-bp fragment of the *PID* promoter encompassing the *cis*-elements (G-box, CACGTG) recognized in vivo by SPT (Extended Data Fig. 9a,d). Moreover, the electrophoresis mobility shift signal was strongly abolished when the wild-type G-box sequence of the *PID* promoter was mutated (TGATGA)^44^ (Extended Data Fig. 9d), in agreement with previous data^19^. Notably, when SPT was incubated with the *PID* promoter fragment in the presence of recombinant 5TPR–SEC–HA and 3TPR–SPY–HA proteins and their respective donor substrates, we observed a stronger mobility shift occurring at lower concentrations of SPT recombinant protein (Fig. 6c).

Altogether, our findings show that SEC and SPY can modulate SPT-mediated control of *PID* expression by increasing its DNA-binding affinity and/or transcriptional activity rather than its ability to form homo- or heterodimers.

## Discussion

This work provides the genetic and molecular foundation for elucidating the role of *Arabidopsis thaliana* SEC^18^ and SPY^26^ *O*-glycosyl enzymes in style development^5,6^. More broadly, our data provide a molecular characterization of the functional roles of *O*-GlcNAc and *O*-fucose PTMs in the regulation of a plant bHLH TF—that is, SPT, which plays pivotal roles in tissue identity^5^, body-axis formation^8^ and bilateral-to-radial symmetry transition^6^ during gynoecium development. As comparable biological processes and conserved molecular players (that is, *O*-GlcNAc^14^ and bHLH TFs^45^) are present in both the plant and animal kingdoms, this study sheds light on a fundamental mechanism that could be employed more generally during the orchestration of organ morphogenesis and symmetry establishment in higher eukaryotes.

Ever since its initial discovery in the 1980s by Torres and Hart^46^, the significance of *O*-GlcNAc as a signalling molecule in processes such as nutritional sensing, stress response and animal development has been long established. Despite its importance in several cellular processes and the extensive range of targets modified by *O*-GlcNAc^19^, the impact of this PTM on protein functions has been comprehensively elucidated for only a limited number of targets. Mechanistic examples that might share similarity to the effect of *O*-GlcNAc on SPT include three well-characterized TFs from the animal kingdom: PDX-1 (ref. ^25^), Oct4 (ref. ^22^) and C/EBPβ^24^.

In plants, it has been previously proposed that SEC and SPY control the activity of other acceptor substrates by promoting protein stability^47^, controlling cellular localization^48^, enhancing or disrupting protein–protein interactions^26,49^ and fine-tuning gene expression^48^. Among these, the best-characterized molecular example is the DELLA protein RGA, which is antagonistically regulated by SEC and SPY during *Arabidopsis* post-embryonic development^18,26^.

Here we showed that the OGT enzyme SEC and the POFUT enzyme SPY can both physically interact with SPT in vivo and in vitro (Fig. 2) and modify several Ser/Thr residues at the N terminus of SPT (Fig. 1 and Extended Data Figs. 1 and 3) flanking its AH domain (Fig. 2c and Extended Data Fig. 4). Mutations of the SPT sequence at Ser23 to Ser25 and Thr71 to Thr74 (to Ala) demonstrated a functional role for those residues in style morphology, thus linking specific *O*-GlcNAc and *O*-fucose modifications to organ function and development.

The AH domain of SPT is quite different from the N-terminal sequence of other plant bHLH TFs^35^, reinforcing the idea that it hosts specific residues for signal transduction mediated by *O*-GlcNAc and *O*-fucose. Notably, the AH domain has been found to be either highly conserved across some SPT orthologues (for example, tomato) or missing from other plant species completely (for example, in monocot rice)^40^. This supports a scenario in which the evolution of the N terminus of SPT and its regulation by sugar decorations might be the site of style/stigma shape diversification in monocots and dicots. It has long been known that the AH domain of SPT supports the essential role of the Ac domain in carpel function^35^, but the molecular basis of its modulation is yet to be determined.

*O*-glycosylation of SPT did not lower its levels, either at the transcriptional or at the protein level (Extended Data Fig. 2c–e), even though *O*-fucosylation of SPT by SPY might, in addition, fine-tune SPT protein stability, as we observed a slight increase of SPT protein levels at the gynoecium apex in the *spy-3* single mutant background (Extended Data Fig. 2d,e). Although the N terminus of SPT is predicted to be unstructured by AlphaFold^50^ (AF-Q9FUA4-F1) and the residues modified by PTMs are located within a disordered region of SPT, we excluded a possible effect of the mutations on protein folding or instability as the C-terminal YFP, cloned in *cis* to the wild-type and mutant versions of SPT, was visible, suggesting that the transgene protein precursors are correctly folded and expressed (Fig. 5b). Since the N-terminal region, including both the AH and Ac domains, is important for SPT transcriptional activation^35^, the attachment of PTMs flanking the AH domain supports two mechanistic scenarios for the function of the AH domain as a modulator of the Ac domain activity: (1) a direct effect via a conformational change or (2) an indirect effect via interaction with other transcriptional activators and/or co-repressors.

While SEC and SPY did not enhance SPT’s ability to form protein–protein interactions with itself and its known dimerization partners IND and HEC1 in tobacco leaves (Fig. 6a), we observed an augmented transcriptional repression of its known downstream target *PID*^6,9^ (Fig. 6b). Accordingly, the effect of glycosylation on SPT increased the affinity of SPT to a DNA fragment of the *PID* locus (Fig. 6c). Altogether, this supports a working model where SEC and SPY, and the PTMs they transfer onto SPT, can directly modify the affinity of SPT for its DNA targets and its transcriptional activity (Fig. 6d). To delve deeper into this scenario, in vivo transcriptomic studies that employ cellular resolution across various gynoecium tissue types will be essential to provide insights into the spatiotemporal contribution of PTMs to SPT activity.

*O*-GlcNAc has been linked to the cold-temperature response in both animals^51^ and plants^31^. Interestingly, in addition to being a master regulator of style development, SPT can transduce environmental cues into developmental programmes by integrating external signals such as cold temperature^52,53^ and light quality^54^ during seed germination and style development. Even though SPT was the first regulatory gene described to control the cold response during seed germination^52^, the molecular mechanism underpinning this process is still unelucidated, and post-translational control of SPT was hypothesized to underpin its mechanistic activity^53^. This raises the intriguing possibility that SPT might be regulated by *O*-GlcNAc at the post-transcriptional level to integrate environmental cues into organ development.

A recent *O*-fucosylomics^55^ study performed in young *Arabidopsis* seedlings identified an N-terminal peptide of SPT modified by *O-*fucose, coinciding with our peptide_1 (Fig. 1), whose modification was lost in the *spy-4* mutant background. This strengthens the importance of sugar-based PTM of SPT in other developmental contexts—for example, roots and cotyledons. Such a regulatory mechanism would fit well with the view of SPT as integrator of abiotic signals and genetic factors, similar to that proposed for the DELLA protein RGA^56^. Conversely, even though 262 *O*-GlcNAc-modified flower proteins have been identified^21^, a gap in knowledge remains regarding the SPY targets and the impact of *O*-fucose on key regulators of gynoecium development. This includes the CK signalling pathway, as implied by the heightened responsiveness of *spy* alleles to CK treatments (Extended Data Fig. 6). The severe phenotype of the *spt* *spy* double mutant is thus in line with a scenario in which other key regulators of style development and hormonal regulators are *O*-fucosylated by SPY in parallel to SPT, leading to the augmented phenotype observed in the *spt* *spy* mutant (Fig. 4).

Notably, the complex network of TFs orchestrating style development includes several layers of cross- and self-regulation that act at both the transcriptional and translational levels^15^. Although our FRET–FLIM experiments in tobacco leaves indicate that SEC and SPY may not regulate SPT–SPT, SPT–IND and SPT–HEC1 dimer formation (Fig. 6a), it is still possible that specific TF interactions might be mediated by *O*-GlcNAc and *O*-fucose in a cell-/tissue-type manner and/or at a specific developmental stage. Also, additional upstream regulation can be hypothesized, as three NGA family members have been identified as targets of *O*-GlcNAc modification in *Arabidopsis* inflorescences^21^.

Our genetic analysis and the phenotypes unveiled by the inducible *spy-3 SEC RNAi* mutant confirm fundamental roles for these two enzymes in post-embryonic growth, including seedling and root growth as well as fruit and style development (Fig. 3). The synergistic role of SEC and SPY in *Arabidopsis* reproductive development has been previously observed by haplo-insufficiency genetic analysis, which revealed that heterozygosis of *SEC/sec-1* in a *spy-4* *ga1* double mutant background increased the number of gynoecia with four carpels^36^, a phenotype previously associated with the *spy-2* allele^57^. In contrast, heterozygosis of *SPY/spy-4* in a *sec-1* *ga1* double mutant background produced pin-like structures^32,36^, which are reminiscent of the *pid* mutant^32^. This is in line with our work showing that SEC and SPY have multiple, overlapping functions in gynoecium development, including tissue specification and style elongation, presumably via the regulation of a plethora of targets, including SPT. It also suggests that the ratio of *O*-GlcNAc and *O*-fucose on target proteins might trigger very specific developmental outputs in vivo. It is possible to speculate that, during style development, different arrays of PTMs on SPT (and other targets), triggered by both genetic and environmental cues, might determine the selectivity of DNA targets and/or protein partners bound by SPT, to specify medial tissue identity^5^, coordinate the medio-lateral^7^ and adaxial–abaxial^8^ body axes and control the antagonistic auxin/CK balance, to establish radial symmetry^6,11,58^.

Taking the data as a whole, we propose a working model (Fig. 6d) in which *O*-glycosylation of SPT by *O*-GlcNAc and *O*-fucose is mediated by SEC and SPY, respectively, to regulate style development by supporting cell elongation and the SPT-mediated balance of the auxin/CK crosstalk at the gynoecium apex.

## Methods

### Plant materials and growth conditions

The following loss-of-function mutant lines in the ecotype Columbia (Col-0) background were used in this study: *sec-2* (ref. ^30^), *sec-5* (ref. ^33^), *spy-3* (ref. ^34^), *ind-2* (ref. ^13^) and *spt-12* (ref. ^59^). The *spy-4* mutant^34^, which was originally in the Wassilewskija background, was back-crossed to Col-0 three consecutive times, and homozygous segregating seeds were used for this study. The *SPT*::*SPT*–*sYFP* transgenic line was described previously^41^. The *SPT*::*SPT*–*sYFP* line in the Col-0 background was crossed with *spt-12* to obtain the complementation line used in this study. Furthermore, the *spt-12/SPT*::*SPT*–*sYFP* line was crossed with *sec-5* and *spy-3* mutants. The plants were grown at 22 °C in long-day conditions (16 h light / 8 h dark) in controlled-environment rooms, unless otherwise specified.

### Immunoprecipitation from *Arabidopsis* inflorescences

*SPT–sYFP* was immune-precipitated from young inflorescences of *spt-12/SPT*::*SPT*–*sYFP* (segregated control), *spt-12;sec-5/SPT*::*SPT*–*sYFP sec-5* and *spt-12;spy-3/SPT*::*SPT*–*sYFP* plants. Young buds (close sepals) were collected from the inflorescences and immediately frozen in liquid N_2_ after picking; 5 g were used in each of the three biological replicates for each genotype. Each sample was ground in liquid N_2_ and extracted using 10 ml of buffer A as previously described^48^. After centrifugation at 16,000 *g* for 30 min, the supernatant was incubated with 30 μl of GFP-Trap magnetic beads (ChromTek). After 1 h of rotation at 4 °C, the beads were sedimented with a magnetic rack (GE Healthcare) for 1 min and washed with buffer A three times. Proteins on the beads were eluted with 1× loading buffer (Merck) and then separated by 10% SDS–PAGE and used for MS analysis.

### MS and data processing

Gel slices were prepared according to standard procedures adapted from Shevchenko et al.^60^. Briefly, the slices were washed with 50 mM TEAB buffer, pH 8 (Sigma), incubated with 10 mM DTT for 30 min at 65 °C and then incubated with 30 mM iodoacetamide at room temperature (both in 50 mM TEAB). After washing and dehydration with acetonitrile, the gels were soaked with 50 mM TEAB containing 10 ng µl^−1^ Sequencing Grade Trypsin (Promega) and incubated at 40 °C for 8 h. The extracted peptide solution was dried down, and the peptides were dissolved in 0.1% TFA/3% acetonitrile. Aliquots were analysed by nanoLC–MS/MS on an Orbitrap Eclipse Tribrid mass spectrometer coupled to an UltiMate 3000 RSLCnano LC system (Thermo Fisher Scientific). The instruments were controlled by the Orbitrap Eclipse Tune Application v.3.4, Thermo Scientific Xcalibur v.4.4.16.14 and the Thermo Scientific SII for Xcalibur v.1.6.0.60983 software. The samples were loaded and trapped using a pre-column with 0.1% TFA at 15 µl min^−1^ for 4 min. The trap column was then switched in-line with the analytical column (nanoEase M/Z column, HSS C18 T3, 100 Å, 1.8 µm; Waters) for separation using the following gradient of solvents A (water, 0.1% formic acid) and B (80% acetonitrile, 0.1% formic acid) at a flow rate of 0.2 µl min^−1^: 0–3 min, 3% B; 3–10 min, increase B to 7% (curve 4); 10–70 min, linearly increase B to 37%; 70–90 min, linearly increase B to 55%, followed by a ramp to 99% B and re-equilibration to 3% B. Data were acquired with the following mass spectrometer settings in positive ion mode: MS1/OT, resolution 120 K, profile mode, mass range *m*/*z* 300–1,800, AGC 4e5, fill time 50 ms; MS2/IT, data-dependent analysis with the following parameters: 1.5 s cycle time in IT turbo mode, centroid mode, isolation window 1 Da, charge states 2–5, threshold 1e4, HCD CE = 33, AGC target 1e4, max. inject time auto, dynamic exclusion 1 count, 15 s exclusion, exclusion mass window ±10 ppm.

Alternatively (for the tobacco sample analysis), the following gradient of solvents A (water, 0.1% formic acid) and B (80% acetonitrile, 0.1% formic acid) was used at a flow rate of 0.2 µl min^−1^: 0–3 min, 3% B; 3–10 min, increase B to 7% (curve 4); 10–100 min, linearly increase B to 37%; 100–148 min, linearly increase B to 50%, followed by a ramp to 99% B and re-equilibration to 3% B. The mass spectrometer settings for those samples were: MS1/OT, resolution 120 K, profile mode, mass range *m/z* 300–1,800, AGC 4e5, fill time 50 ms; MS2/IT, data-dependent analysis with the following parameters: FAIMS device set to three compensation voltages (−35V, −50V and −65V) for 1 s each; MS2/IT: for each CV, data-dependent analysis with the following parameters: IT turbo mode, centroid mode, isolation window 1 Da, charge states 2–5, threshold 1e4, HCD CE = 30, AGC target 1e4, max. inject time dynamic, dynamic exclusion 1 count, 15 s exclusion, exclusion mass window ±10 ppm.

LC–MS analysis using EThcD fragmentation was performed on an Orbitrap Fusion Tribrid mass spectrometer (Thermo Fisher Scientific) using similar basic parameters as described above. Specific parameters for EThcD fragmentation included: targeted inclusion of the peptides of interest with ±25 ppm tolerance, ETD active with calibrated charge dependent parameters, EThcD true with hcd25, detector type: ion trap rapid.

The raw data from all acquisitions were processed in Proteome Discoverer v.2.4 or v.3.0 (Thermo Scientific); all mentioned tools of the following workflow are nodes of the Proteome Discoverer software. Spectra were recalibrated, and identification was performed using an in-house Mascot Server v.2.8.0 to v.2.8.2 (Matrixscience) with the TAIR10_pep_20101214 *A. thaliana* protein sequence database (arabidopsis.org, 35,386 entries) or the *Nicotiana tabacum* database^61^ to which the SPT–YFP fusion sequence was added. The MaxQuant contaminants database (maxquant.org, 245 entries) was included in the search. The parameters were enzyme trypsin, 2 missed cleavages, 6 ppm precursor tolerance, 0.6 Da fragment tolerance, carbamidomethylation (C) as fixed modification and oxidation (M), deamidation (N/Q), acetylation (protein N terminus), dHex (NST, +146.058 Da) and HexNAc (NST, +203.079 Da) as variable modifications. Evaluation was performed using Percolator with an FDR target of 0.01.

For processing the EThcD data, Proteome Discoverer v.3.0 with Mascot Server v.2.8.0 set to EThcD was used with a custom database containing the SPT sequence and common contaminants. The parameters were enzyme trypsin, 1 missed cleavage, 10 ppm precursor tolerance, 0.6 Da fragment tolerance, carbamidomethylation (C) as fixed modification and dHex (NST, +146.058 Da) and HexNAc (NST, +203.079 Da) as variable modifications. Evaluation was performed using Fixed Value PSM Validator with a maximum delta Cn of 0.05.

For all data, the Minora Feature Detector was used for peak detection and quantification with a minimum trace length of 7 and S/N 3. Peptide abundances were determined as peak areas. After normalization to the total peptide amount, the quantification was based on the top three unique peptides per protein. Missing values were imputed by low abundance resampling. For hypothesis testing, a background-based *t*-test was applied. The results were exported to Microsoft Excel. The percentage of the peptide modified with *O*-GlcNAc or *O*-fucose was calculated on the basis of the normalized abundances of the modified peptide compared with the sum of the abundances of all versions of the peptide.

For overviews and spectrum presentation, the MS raw files were converted to .mgf files using msconvert (Proteowizard v.3, https://proteowizard.sourceforge.io/index.html); these files were used for a database search via Mascot Server v.2.8.0 to v.2.8.2 using the abovementioned databases and Mascot search parameters. The Mascot search results were then imported into Scaffold v.4.11.0 (www.proteomesoftware.com).

### DNA constructs

To produce the recombinant 6xHis–SPT protein, the full-length *SPT* coding sequence was cloned into the *pRSFDuet* vector by using the BamHI and PstI restriction sites. To produce recombinant 5TPR–SEC and 3TPR–SPY proteins, we used the following strategy. First, to generate a protein expression vector with the 10xHis–MBP tag, we amplified a *10xHis–MBP* coding fragment by PCR with the primer pair 10xHis–MBP-F and 10xHis–MBP-R from the *pMAL–c2X* vector (Addgene) and cloned it into the *pTrcHis* vector (Addgene) using the XhoI and HindIII restriction enzymes, obtaining a *pTrc10xHis–MBP* vector. Second, code-optimized full-length *SEC* and *SPY* coding sequences were synthesized by Sangon Biotech (Shanghai). The coding sequences of *5TPR–SEC* and *3TPR–SPY* were amplified using the gene-specific primers pair 5TPR–SEC–opt_F/5TPR–SEC–opt_R and 3TPR–SPY–opt_F/3TPR–SPY–opt_R and cloned into the *pTrc10xHis–MBP* vector using the SacI and SalI restriction enzymes. All constructs were verified by sequencing and transformed into the *Escherichia coli* Rosetta (DE3) strain by heat shock.

For transient co-expression in tobacco, *35S*::*SPT–3xFLAG*, *35S*::*SEC–3xHA* and *35S*::*SPY–3xHA* were constructed as follows: the full-length coding sequences of *SPT*, *SEC* and *SPY* were amplified using gene-specific primers (listed in Supplementary Table 3), and the PCR products were digested with SifI (NEB) and cloned into the empty *pCambia1305–35S*::*3xFLAG* or *pCambia1305–35S*::*3xHA* vector (gifted by Y. Ding), which was pre-digested with DraIII (NEB). All constructs were verified by sequencing and introduced into the *Agrobacterium tumefaciens GV3101* strain for infiltration in *N. benthamiana* leaves.

For the split-luciferase complementation assay, the coding sequences of *SPT*, *SEC* and *SPY* were amplified by PCR using gene-specific primers (listed in Supplementary Table 3) and inserted into the *pCambia1305–35S*::*nLuc* and *pCambia1305–35S*::*cLuc* vectors (gifted by Y. Ding), respectively, using DraIII restriction sites, thus producing the *35S*::*SPT–nLuc*, *35S*::*SEC–cLuc* and *35S*::*SPY–cLuc* constructs.

For the Y2H assay, full-length and domain fragments of coding sequences from SPT, SEC and SPY were cloned into *pDONR221* (Invitrogen) using primers in Supplementary Table 3, and then recombined into either the *pGDAT7* or *pGBKT7* vector (Clontech) following the manufacturer’s instructions.

The DEX-inducible amiRNA constructs for *SEC* were generated as follows: specific amiRNA primers were generated using the online tool ‘Web MicroRNA Designer’ (WMD3) (http://wmd3.weigelworld.org/cgi-bin/webapp.cgi)^62^. According to WMD3 guidelines, the amiRNAs were designed with uridine at position 1; in addition, one mismatch to the target gene was introduced in the 3′ part of the amiRNAs to reduce the likelihood that the amiRNA would act as a primer for RNA-dependent RNA polymerases to trigger secondary RNAi. Two specific regions, ‘ACGTGCAACCTTCTACACACC’ (named *a1SEC*) and ‘CTGGGCTCCCTGTAAAACGTG’ (named *a2SEC*), located in the 15th and 18th exons of the *SEC* gene, respectively, were chosen (Extended Data Fig. 5). Using pRS300 (ref. ^62^) as a PCR template, the fragments were amplified, and subsequently each fragment was inserted downstream of a DEX-inducible promoter to generate two *pGAL6*::*amiRNA* plasmids. The resultant plasmids were recombined with the *35S*::*GVG*::*Nos* cassette (*pICSL11041*, Synbio TSL) and the Basta selection marker cassette (*pICH41308*, Synbio TSL), using the standard Golden Gate cloning method, to produce the binary vectors *p35S*::*GVG–amiSEC–Basta–pICSL4723* (*p35S*::*ami1SEC–GR* and *p35S*::*ami2SEC–GR*). The vectors were transformed into the *spy-3* mutant background.

To generate the transgenic *SPT–YFP* point-mutation lines analysed in this study, we amplified a 5-kb *SPT* promoter fragment from Col-0 genomic DNA using the primer pair pSPT-F and pSPT-R (see Supplementary Table 3 for the sequences). The *SPT* promoter was cloned into the *pCambia1305* vector by using the EcoRI and BspEI restriction enzymes, thus producing the *pSPT–pCambia1305* plasmid. The *SPT* wild-type genomic coding sequence fused in frame to the *sYFP* fluorescent tag (hence named *SPT–YFP*) was then amplified as a whole segment (2.5 kb) from genomic DNA extracted from original *SPT*::*SPT*–*sYFP*^48^ seedlings using the primer pair 2.5k_(BspEI)_F and 2.5k_(KpnI)_R and cloned into the *pSPT–pCambia1305* plasmid, thus obtaining the *pSPT*::*SPT–YFP–pCambia1305* construct.

To generate constructs harbouring specific point mutations of SPT, we used the *pSPT*::*SPT–YFP–pCambia1305* plasmid as a template and used mutagenesis primers (listed in Supplementary Table 3) introducing specific mutations in the *SPT* coding sequence. All constructs were verified by sequencing and introduced into the *A. tumefaciens GV3101* strain by heat shock, then transformed into the *spt-12* background by floral dipping (note, since *spt-12* produces few seeds, each construct was transformed in 30 mutant plants). The transgenic seeds were selected on Murashige and Skoog plates supplied with 15 mg l^−1^ Basta, and at least eight positive, independent T_1_ lines were screened using a confocal microscope for the presence of nuclear YFP signals in roots.

For the FRET–FLIM assay, the coding sequences of *SPT* and *IND* were first inserted into *pCambia1305* using the DraIII sites; then, the coding sequences of *EGFP* and *RFP* were inserted in-frame using the XbaI and PstI restriction enzymes.

For the transactivation assay, we first cloned the *GUS* coding sequence into the *pCambia1305* vector using the HindIII and BstEII sites, producing the *pCambia1305–GUS* construct. The 1-kb promoter region of *PID* was then cloned into the *pCambia1305–GUS* vector using the PstI and HindIII restriction sites. All constructs were verified by sequencing and introduced into the *A. tumefaciens GV3101* strain for transformation in *N. benthamiana* leaves.

### Recombinant protein expression

Cells were grown in LB media at 37 °C until the OD_600_ value reached 0.5; the culture was then cooled down before induction. The expression of *6xHis–SPT* was induced by 0.4 mM IPTG at 25 °C for 4 h. The expression of *10xHis–MBP–5TPR–SEC* and *10xHis–MBP–3TPR–SPY* was induced by 0.4 mM IPTG at 16 °C for 16 h. Recombinant proteins were purified with nickel sepharose according to the manufacturer’s instructions (QIAGEN). The purified recombinant protein was dialysed overnight against dialysis buffer (20 mM Tris-HCl, pH 8.0, 50 mM NaCl, 1 mM DTT, 5% glycerol) at 4 °C. All proteins were aliquoted, flash-frozen in liquid N_2_ and stored at −80 °C.

### In vitro *O*-glycosylation assay

The direct modification of SPT protein by SEC and SPY was tested by an in vitro glycosylation assay as previously described in Zentella et al.^26^. Briefly, to test *O*-GlcNAclation, a 50 ml reaction was carried out by mixing 10 mg of 6xHis–SPT, 5 mg of 5TPR–SEC, 20 mM Tris-HCl (pH 7.2), 12.5 mM MgCl_2_ and 200 μM UDP–GlcNAc (Merck). To test *O*-fucosylation, the 50 ml reaction contained 10 mg of 6xHis–SPT, 5 mg of 3TPR–SPY, 50 mM Tris-HCl (pH 8.2), 50 mM NaCl, 5 mM MgCl_2_ and 200 μM GDP–fucose (Merk). After incubation for 2 h at 25 °C, the protein samples were separated by 10% SDS–PAGE, and the band containing recombinant 6xHis–SPT (43 kDa) was excised and treated for MS analysis as described above.

### Co-IP assay in tobacco leaves

*A. tumefaciens GV3101* strains harbouring the *35S*::*SPT–3xFLAG*, *35S*::*SEC–3xHA* and *35S*::*SPY–3xHA* constructs were transiently expressed either alone or co-infiltrated in four-week-old leaves of *N. benthamiana*. To enhance gene expression, an *Agrobacterium* strain harbouring P19 was always co-infiltrated. After 48 h from infiltration, the leaves were harvested, and the total protein was extracted using the protein extraction buffer (25 mM Tris-HCl, pH 7.4, 1 mM EDTA, 150 mM NaCl, 10% glycerol, 0.15% NP-40, 1 mM NaF, 10 mM DTT, 2% PVPP, 1 mM PMSF and 1× protein inhibitor). Total protein extracts were immunoprecipitated with anti-HA magnetic beads (Thermo Scientific); input and IP samples were analysed by immunoblotting using anti-HA (Sigma) and anti-FLAG antibodies (Sigma) separately.

### Split-luciferase complementation assay in tobacco leaves

The split-luciferase complementation assay was performed as described previously^55^. *A. tumefaciens GV3101* strains harbouring the *35S*::*SEC–cLuc* and *35S*::*SPY–cLuc* constructs were co-infiltrated with either *35S*::*SPT–nLuc* or a *35S*::*Ø–nLuc* empty vector, while *35S*::*SPT–nLuc* was also co-infiltrated with a *35S*::*Ø–cLuc* empty vector, into four-week-old leaves of *N. benthamiana*. To enhance gene expression, an *Agrobacterium* strain harbouring P19 was always co-infiltrated. After 48 h infection at room temperature, 0.4 mM d-luciferin (ThermoFisher) was infiltrated into the leaves, and LUC activity was measured using the NightOWL system equipped with a cooled CCD imaging apparatus (Berthold Technologies) and analysed with the Indigo software (v.2.0.5.0).

### Y2H assay

For the Y2H experiments, coding DNA sequences were cloned into pDONR207 and recombined into *pGDAT7* or *pGBKT7* vectors (Clontech). Plasmids were transformed into the AH109 yeast strain by the lithium acetate method. Co-transformed strains were selected on SD/−Leu/−Trp (Merk) at 28 °C for three to four days. Transformed yeast were serially diluted (10^0^, 10^−1^, 10^−2^ and 10^−3^) and dotted on SD/−Ade/−His/−Leu/−Trp and SD/−Ade/−His/−Leu/−Trp/ 2.5 mM 3-amino-1,2,4-triazole (3-AT, Merk) to examine protein interactions. Growth was observed after five days of incubation at 28 °C.

### Gynoecium treatments

For the DEX treatments, *35S*::*amiSEC–GR* inflorescences were sprayed with either 10 µM DEX (Sigma) or mock (DMSO, Sigma) three times every five days over two weeks, and gynoecia were fixed after five days from the last treatment. For the CK (6-benzylaminoadenine, BA) treatments, the inflorescences of Col-0, *sec-2*, *sec-5*, *spy-3* and *spy-4* were sprayed with either 50 µM or 100 µM BA (Merk) or mock (NaOH (200 mM)) three times every five days over two weeks, and gynoecia were fixed after five days from the last treatment. All spray treatments used a 0.015% final concentration of Silwet L-77.

### SEM

Inflorescences were fixed overnight in FAA solution (3.7% formaldehyde, 5% glacial acetic acid and 50% ethanol). After complete dehydration through an ethanol series from 50% to 100%, the inflorescences were critical point dried using the Leica EM CPD300. Gynoecia were hand-dissected using a stereomicroscope (Leica S9D), coated with gold particles and examined with an FEI Nova NanoSEM 450 emission scanning electron microscope equipped with xT microscope control software (v.6.3.4), using an acceleration voltage of 3 kV. The experiments were conducted in biological triplicates, and gynoecia were taken from distinct inflorescences and plants each time. The total number of gynoecia analysed for each experiment is indicated in the figure legends. Data analysis was carried out using Microsoft Excel (v.2311).

### Confocal microscopy

Confocal microscopy analysis was performed on a Zeiss LSM 880 confocal microscope with a ×40 water-immersion lens, using ZEN-Black-LSM880 software (v.2.3) (Zeiss). YFP signals were excited by an argon-514 nm, 10 mW solid laser with emission at 550–570 nm. To view the gynoecia, floral buds were dissected using a stereomicroscope (Leica S9D); then, the gynoecia were mounted along their longitudinal axis in water. To quantify the YFP fluorescence intensity, the *z*-series images of epidermal cells on the surface layer (L1) at the style region were collected with the *z*-step set at 1 μm. Maximum intensity projection of the *z*-series of the nucleus was used to quantify the YFP intensity using the software ImageJ (v.1.53).

### RNA extraction and qRT-PCR

Three independent biological repeats of total RNA extracted from each genotype were isolated from young inflorescences using an RNeasy Plant Mini Kit (Qiagen), including treatment with RNase-free DNase (Qiagen) following the manufacturer’s instructions. Four micrograms of extracted RNA sample was reverse transcribed using M-MLV Reverse Transcriptase (Promega) from each RNA sample. The qRT-PCR experiments were performed in quadruplicates using SYGREEN BLUE qPCR MIX (PCRBIO) with a Chromo4 Real-Time PCR Detection System (Bio-Rad). Target gene expression levels were quantified by the 2^−ΔΔct^ method with *UBIQUITIN10* as the internal control using Bio-Rad CFX manager software (v.3.1) and Microsoft Excel (v.2311). The gene-specific primers are listed in Supplementary Table 3.

### Western blot analysis

Protein samples were separated by 10% SDS–PAGE and transferred to a nitrocellulose membrane (GE Healthcare). After being blocked in 1× PBST buffer containing 5% skimmed milk, the membrane was incubated with the selected primary antibody using a 1,000-fold dilution overnight at 4 °C, washed three times with 1× PBST (10 min each) and incubated with the selected secondary antibody conjugated with horseradish peroxidase using a 3,000-fold dilution for 1 h at room temperature. After three washes with 1× PBST (10 min each), the film was illuminated and photographed with ImageQuant800 (GE Healthcare). AMERSHAM ImageQuot 800 software (v.1.2.0) was used for western blotting analysis; GeneSys software (v.1.3.8.0) was used for gel imaging. If the primary antibodies were already conjugated with horseradish peroxidase, there was no need to incubate the membrane with secondary antibodies. The following antibodies were used: anti-GFP (GF28R, 1:1,000 dilution, Thermo Scientific), anti-RFP (ab34771, 1:2,500 dilution, Abcam), anti-FLAG (F3165, 1:3,000 dilution, Sigma), anti-HA (3F10, 1:3,000 dilution, Sigma), anti-mouse (sc-516102, 1:1,000 dilution, Santa Cruz) and anti-rabbit (ab205718, 1:5,000 dilution, Abcam).

### Seed germination assay

Freshly harvested seeds were sterilized and sowed on 0.9% agar plates supplemented with 10 µM DEX or mock (DMSO). The plates were grown in long-day (16 h light / 8 h dark) conditions. Seed germination was photographed from day 0 for three consecutive days, and the rate of germination was calculated as the ratio of germinated seeds over the total number of seeds plated, for each genotype every 24 hours. Data analysis was carried out using Microsoft Excel (v.2311).

### Root length measurement

Seeds were sterilized and stratified in a dark, cold room for 48 h before being sowed on Murashige and Skoog plates supplemented with 10 µM DEX or mock (DMSO). The seed plates were grown vertically (with a slight angle) in short-day (8 h light / 16 h dark) conditions. The seedlings were imaged at day 7 after germination, and the primary root lengths were measured using ImageJ (v.1.53).

### FRET–FLIM assay

FLIM images were captured using a Leica Stellaris 8 FALCON confocal microscope equipped with a ×40 water-immersion objective (HC Plan 40x/NA 1.10). The samples were excited with the 488 nm output of a pulsed white-light laser working at a 20 MHz repetition rate (Pulsed SuperK Fianium FIB-12 PP white-light laser from NK Photonics). The full width at half maximum of the laser pulse was ~170 ps, as determined from the instrument response function that was obtained by imaging an Erythrosin B (Sigma-Aldrich, >95% purity) water solution saturated with potassium iodide (Sigma-Aldrich). The EGFP fluorescence signal was collected in the 503–530 nm range using a Leica HyD X detector. Four-week-old *N. benthamiana* leaves were co-infiltrated with *GV3101* strains to express *SPT–EGFP* with *RFP–NLS* or in combination with *IND–RFP*, *HEC1–RFP* and *SPT–RFP*, with or without *SEC–3xHA* and *SPY–3xHA* recombinant proteins. To enhance gene expression, an *Agrobacterium* strain harbouring P19 was always co-infiltrated. After 48 h, the leaves were cut and imaged on the microscope. Image acquisition and analysis was carried out using LAS X software (v.4.2, Leica Microsystems). At least 50 nuclei per condition from three biological replicates were analysed. A region of interest was hand-drawn around the nucleus, and the arrival times of all photons within the region of interest were used to generate a fluorescence decay histogram. The fluorescence decay was fitted using a bi-exponential decay function, and the amplitude-weighted average fluorescence lifetime^63^ obtained from the fit was used to compute the energy transfer efficiency, *E*, in accordance with *E* = 1 − *τ*_DA_/*τ*_D_ (ref. ^64^), where *τ*_DA_ and *τ*_D_ are, respectively, the average fluorescence lifetimes of EGFP in the presence and absence of the FRET acceptor, RFP. The latter fluorescence lifetime, *τ*_D_, was obtained from the negative control, SPT–EGFP::RFP–NLS.

### Chromatin immunoprecipitation–qPCR assay

The chromatin immunoprecipitation assay was performed using young inflorescences (close sepals) of the *SPT–sYFP spt-12* complementation line as previously described^65^. Three biological repeats were performed, using 3 g of plant material for each repeat. IP was conducted using GFP-Trap beads (ChromTek). The enrichment of the *PID* promoter regions was quantified using qPCR normalized with the *ACTIN2* gene with the appropriate primers listed in Supplementary Table 3.

### Transient transactivation assay

Transactivation assays were performed on four-week-old *N. benthamiana* *Agrobacterium*-infiltrated leaves using *pPID*::*GUS* alone, *pPID*::*GUS* combined with *35S*::*SPT–GFP* and *35S*::*IND–RFP* or together with *35S*::*SEC–3xHA, 35S*::*sec-2–3xHA, 35S*::*SPY–3xHA* or *35S*::*spy-3–3xHA*. To enhance gene expression, an *Agrobacterium* strain harbouring P19 was always co-infiltrated. All transformed bacteria were infiltrated using OD = 0.5, except *35S*::*SPT–GFP* and *35S*::*IND–RFP* (Fig. 6b), which were infiltrated using OD = 0.1. After 48 h from infiltration, the leaves were harvested, and the expression levels of *GUS* transcript were quantified via qRT-PCR; the *hygromycin* gene in the *pPID*::*GUS* vector was used as the internal control. Protein expression levels were detected by western blot. The primers used are listed in Supplementary Table 3.

### EMSA

The EMSA reaction was performed as follows: 150 ng of amplified and gel-extracted wild-type or mutated DNA nucleotides together with the indicated concentration of recombinant 6His–SPT protein were incubated in 1× EMSA buffer (250 mM Tris-HCl, pH 8.0, 500 mM NaCl, 25% glycerol, 10 mM DTT) on ice for 20 min. To test the effect of *O*-glycosylation on SPT, recombinant SPT was first incubated with 5TPR–SEC or 3TPR–SPY recombinant proteins in reaction buffer for 1 h at 25 °C with 200 µM UDP–GlcNAc or GDP–fucose, respectively (enzymatic assay). Then, the DNA nucleotides and EMSA buffer were added to the reaction. The reaction was analysed by electrophoresis on 5% native acrylamide gel in 1× TBE buffer at 150 V for 50 min. After electrophoresis, the gels were stained with EB for 20 min followed by imaging with the UV imager (G:BOX F3 gel doc system, SYNGENE). The primers used for amplification of the wild-type and mutated *PID* 171-nucleotide fragments are listed in Supplementary Table 3.

The company names and catalogue numbers of the commercial reagents used in this study are summarized in Supplementary Table 4.

### Statistical analysis

Statistical analysis was performed as indicated in each figure legend, using GraphPad Prism v.9 (Dotmatics). The exact *P* values are provided in the ‘Statistical source data1’ file.

### Reporting summary

Further information on research design is available in the Nature Portfolio Reporting Summary linked to this article.

### Supplementary information

Reporting Summary

Supplementary Table 1 EThcD MS proteomics analysis results for peptide_1 in SPT (LISSSSSSSVYDTR) in vivo.

Supplementary Table 2 EThcD MS proteomics analysis results for peptide_2 in SPT (SSPLPSYYSPATTTTTASLIGVHGSGDPHADNSR) in vivo.

Supplementary Table 3 Sequences and purposes for all primers used in this study.

Supplementary Table 4 Company names and catalogue numbers of commercial reagents used in this study.

### Source data

Source Data Figs. 1 and 3–6 and Extended Data Figs. 1, 2, 5 and 7–9 Statistical source data for Figs. 1 and 3–6 and Extended Data Figs. 1, 2, 5 and 7–9.

Source Data Figs. 2 and 6 and Extended Data Fig. 8 Unprocessed western blots and/or gels for Figs. 2 and 6 and Extended Data Fig. 8.

## Data Availability

All data needed to evaluate the conclusions in this paper are present in the paper and/or its supplementary materials. All raw proteomic data are available in the PRIDE repository (https://www.ebi.ac.uk/pride/; accession numbers: PXD037917 (in vivo HCD MS/MS from *A. thaliana*); PXD043987 (in vivo EThcD MS/MS from *A. thaliana*); PXD043957 (in vivo HCD MS/MS from *N. benthamiana*); and PXD044034 (in vitro HCD MS/MS from enzymatic assay)). The following databases were used in this study: TAIR10_pep_20101214 *Arabidopsis thaliana* protein sequence database (arabidopsis.org); the *N. tabacum* database^61^, to which the SPT–YFP fusion sequence was added; and the MaxQuant contaminants database (maxquant.org). All new genetic material (high-order mutants and transgenic lines) and expression vectors will be made available to the scientific community upon request and with no limitation. Source data are provided with this paper.
