# Supplementary material for: O-glycosylation of the transcription factor SPATULA promotes style development in Arabidopsis
Source: Nat Plants. 2024 Jan 26;10(2):283–99. doi: 10.1038/s41477-023-01617-4 (PMC10881398; doi:10.1038/s41477-023-01617-4)
Supplement: Supplementary file 1 — Reporting Summary [file 41477_2023_1617_MOESM1_ESM.pdf]

nature portfolio

Corresponding author(s): Laila Moubayidin

Last updated by author(s): Dec 12, 2023

# Reporting Summary

Nature Portfolio wishes to improve the reproducibility of the work that we publish. This form provides structure for consistency and transparency in reporting. For further information on Nature Portfolio policies, see our Editorial Policies and the Editorial Policy Checklist.

## Statistics

For all statistical analyses, confirm that the following items are present in the figure legend, table legend, main text, or Methods section.

| n/a | Confirmed | |
|---|---|---|
| ☐ | ☒ | The exact sample size (*n*) for each experimental group/condition, given as a discrete number and unit of measurement |
| ☐ | ☒ | A statement on whether measurements were taken from distinct samples or whether the same sample was measured repeatedly |
| ☐ | ☒ | The statistical test(s) used AND whether they are one- or two-sided<br>*Only common tests should be described solely by name; describe more complex techniques in the Methods section.* |
| ☐ | ☒ | A description of all covariates tested |
| ☒ | ☐ | A description of any assumptions or corrections, such as tests of normality and adjustment for multiple comparisons |
| ☐ | ☒ | A full description of the statistical parameters including central tendency (e.g. means) or other basic estimates (e.g. regression coefficient) AND variation (e.g. standard deviation) or associated estimates of uncertainty (e.g. confidence intervals) |
| ☐ | ☒ | For null hypothesis testing, the test statistic (e.g. *F*, *t*, *r*) with confidence intervals, effect sizes, degrees of freedom and *P* value noted<br>*Give P values as exact values whenever suitable.* |
| ☒ | ☐ | For Bayesian analysis, information on the choice of priors and Markov chain Monte Carlo settings |
| ☒ | ☐ | For hierarchical and complex designs, identification of the appropriate level for tests and full reporting of outcomes |
| ☒ | ☐ | Estimates of effect sizes (e.g. Cohen's *d*, Pearson's *r*), indicating how they were calculated |

*Our web collection on statistics for biologists contains articles on many of the points above.*

## Software and code

Policy information about availability of computer code

| | |
|---|---|
| Data collection | xT microscope Control v6.3.4 (for scanning electron microscope); ZEN-Black-LSM880 software (v2.3) (for confocal microscopy); LAS X (v. 4.2, Leica Microsystems) (for FRET-FLIM experiments); AMERSHAM ImageQuot 800 software v1.2.0 (for western blotting); Indigo software (v2.0.5.0) (for Luciferase assay); GeneSys software v1.3.8.0 (for gel imaging); Chromo4 Real-Time PCR Detection System (Bio-Rad) (for qRT-PCR); Orbitrap Eclipse Tune Application v3.4 and Thermo Scientific Xcalibur v4.4.16.14 (for Mass Spectrometry); Thermo Scientific SII for Xcalibur v1.6.0.60983 (for Liquid Chromatography). |
| Data analysis | LAS X (v. 4.2, Leica Microsystems) (for FRET-FLIM experiments); Bio-Rad CFX manager v3.1 (for qRT-PCR); Proteome Discoverer 2.4 and 3.0 (Thermo Fisher Scientific, Hemel Hempstead, UK); Mascot Server 2.8.0 to 2.8.2 (Matrixscience, London, UK); Scaffold 4.11.0 (Proteomesoftware, Portland, USA); Proteowizard (v3) (msconvert tool) (https://proteowizard.sourceforge.io/index.html, Palo Alto, USA); Microsoft excel (v2311); Image J (v1.53); Graphpad Prism9 (v9.5.1); Web MicroRNA Designer' (WMD3, v3.2) |

For manuscripts utilizing custom algorithms or software that are central to the research but not yet described in published literature, software must be made available to editors and reviewers. We strongly encourage code deposition in a community repository (e.g. GitHub). See the Nature Portfolio guidelines for submitting code & software for further information.

## Data

Policy information about availability of data

All manuscripts must include a data availability statement. This statement should provide the following information, where applicable:

- Accession codes, unique identifiers, or web links for publicly available datasets
- A description of any restrictions on data availability
- For clinical datasets or third party data, please ensure that the statement adheres to our policy

All data needed to evaluate the conclusions in this paper are present in the paper and/or the Supplementary Materials. All raw proteomic data are available on the PRIDE repository (https://www.ebi.ac.uk/pride/, accession numbers: PXD037917 (In vivo HCD MS/MS from A.thaliana | Username: reviewer_pxd037917@ebi.ac.uk | Password: bJWpclLH); PXD043987 (In vivo EThcD MS/MS from A.thaliana | Username: reviewer_pxd043987@ebi.ac.uk | Password: qI1fLCOw); PXD043957 (In vivo HCD MS/MS from N.benthamiana | Username: reviewer_pxd043957@ebi.ac.uk | Password: mgdRWFsA); PXD044034 (In vitro HCD MS/MS from enzymatic assay | Username: reviewer_pxd044034@ebi.ac.uk | Password: JPlE48j2). All new genetic material (high-order mutants, transgenic lines) and expression vectors will be made available to the scientific community upon request and with no limitation.

## Research involving human participants, their data, or biological material

Policy information about studies with human participants or human data. See also policy information about sex, gender (identity/presentation), and sexual orientation and race, ethnicity and racism.

| | |
|---|---|
| Reporting on sex and gender | n/a |
| Reporting on race, ethnicity, or other socially relevant groupings | n/a |
| Population characteristics | n/a |
| Recruitment | n/a |
| Ethics oversight | n/a |

Note that full information on the approval of the study protocol must also be provided in the manuscript.

# Field-specific reporting

Please select the one below that is the best fit for your research. If you are not sure, read the appropriate sections before making your selection.

☒ Life sciences ☐ Behavioural & social sciences ☐ Ecological, evolutionary & environmental sciences

For a reference copy of the document with all sections, see nature.com/documents/nr-reporting-summary-flat.pdf

# Life sciences study design

All studies must disclose on these points even when the disclosure is negative.

| | |
|---|---|
| Sample size | Sample-size calculation was not performed. Sample size was chosen following general practice from previous studies and considered sufficient according to statistical significance of the test obtained. |
| Data exclusions | No data were excluded from the analyses. |
| Replication | Unless otherwise specified (i.e., ED_Fig7a), all experiments were performed in biological triplicates, consistently demonstrating reproducible results. In ED_Fig7a we used three independent biological transgenic plants per construct and performed technical replicates. |
| Randomization | In each independent repeat, the biological samples were collected, treated, and analysed randomly, without any bias. The experiments were carried out including randomization such as: independent biological materials, reagents, instruments and participants. |
| Blinding | Blind experiments were performed for Yest-two-hybrid, expression analysis by qRT-PCR and GUS staining, phenotypic analysis (SEM) and confocal analysis. Blinding was performed as much as possible also during sample preparation and dissection stage, where different authors carried out experiments without knowing the identity of the sample under investigation. |

# Reporting for specific materials, systems and methods

We require information from authors about some types of materials, experimental systems and methods used in many studies. Here, indicate whether each material, system or method listed is relevant to your study. If you are not sure if a list item applies to your research, read the appropriate section before selecting a response.

## Materials & experimental systems

| n/a | Involved in the study |
| --- | --- |
| ☐ | ☒ Antibodies |
| ☒ | ☐ Eukaryotic cell lines |
| ☒ | ☐ Palaeontology and archaeology |
| ☒ | ☐ Animals and other organisms |
| ☒ | ☐ Clinical data |
| ☒ | ☐ Dual use research of concern |
| ☐ | ☒ Plants |

## Methods

| n/a | Involved in the study |
| --- | --- |
| ☒ | ☐ ChIP-seq |
| ☒ | ☐ Flow cytometry |
| ☒ | ☐ MRI-based neuroimaging |

# Antibodies

Antibodies used

anti-GFP (GF28R, 1:1000 dilution, Thermo Scientific),
anti-RFP (ab34771, 1:2500 dilution, Abcam),
anti-FLAG (F3165, 1:3000 dilution, Sigma),
anti-HA (3F10, 1:3000 dilution, Sigma),
anti-mouse (sc-516102, 1:1000 dilution, Santa Cruz),
anti-rabbit (ab205718, 1:5000 dilution, Abcam).

Validation

The antibodies were validated in plants (including Arabidopsis).
GFP Monoclonal Antibody Thermo Scientific GF28R; used in Arabidopsis https://nph.onlinelibrary.wiley.com/doi/full/10.1111/nph.12364.
Biotin anti-RFP antibody abcam ab34771; used in Arabidopsis https://www.nature.com/articles/s41467-020-19343-2.
anti-mouse antibody (m-IgGκ BP-HRP) Santa Cruz sc-516102; used in Arabidopsis https://www.mdpi.com/1422-0067/21/24/9666.
Goat Anti-Rabbit IgG H&L (HRP) abcam ab205718; used in Arabidopsis https://bsppjournals.onlinelibrary.wiley.com/doi/full/10.1111/mpp.13037.
Anti-HA-Biotin Merck 3F10; used in Arabidopsis https://www.nature.com/articles/ncomms5848#Sec2.
Monoclonal ANTI-FLAG® M2 antibody Merck F3165; used in Arabidopsis https://www.nature.com/articles/s41477-022-01303-x.
