# Supplementary material for: O-glycosylation of the transcription factor SPATULA promotes style development in Arabidopsis
Source: Nat Plants. 2024 Jan 26;10(2):283–99. doi: 10.1038/s41477-023-01617-4 (PMC10881398; doi:10.1038/s41477-023-01617-4)
Supplement: Supplementary file 4 — Sequences and purposes for all primers used in this study. [file 41477_2023_1617_MOESM4_ESM.pdf]

Primer	Sequence, 5'-3'	Purpose
SPT QPCR F	GATTCGACCCCCTGAAGCAA	qRT-PCR
SPT QPCR R	CTTCCCGACTCATCTCCACG	qRT-PCR
SEC QPCR F	ACCATGCGTTGGGTGAAGAA	qRT-PCR
SEC QPCR R	CCGGGTCCTCATGTGTTTCA	qRT-PCR
SPY QPCR F	CTTGGGAAGTCGTTAGGTGGT	qRT-PCR
SPY QPCR R	AGGTAAAGGCACCGAAAAGC	qRT-PCR
UBQ10_RT_F	GGCCTTGTATAATCCCTGATGAATAAG	qRT-PCR
UBQ10_RT_R	AAAGAGATAACAGGAACGGAAACATAGT	qRT-PCR
qpcr-GUS-F	TTATGCGGGCAACGTCTG	qRT-PCR
qpcr-GUS-R	CCCACACTTTGCCGTAATG	qRT-PCR
qpcr-Hyg-F	GGCGAAGAATCTCGTGCTTT	qRT-PCR
qpcr-Hyg-R	CCGATGCAAAGTGCCGATAA	qRT-PCR
pPID R1-F	GGTCTCTGTAACGTCTTTTTACTG	CHIP-qPCR
pPID R1-R	GATGAAACAGGACAGGGTGTC	CHIP-qPCR
pPID R2-F	GAATCACTAGTCCAATATTTCTCC	CHIP-qPCR
pPID R2-R	CAGTAAAAAGACGTTACAGAGACC	CHIP-qPCR
pPID R3-F	CCTTTAGAAAATTGATGTTATCATTAAAC	CHIP-qPCR
pPID R3-R	GGAGAAATATTGGACTAGTGATTC	CHIP-qPCR
ACTIN11 CHIP-F	CCAATCGTGAGAAAATGACTCAG	CHIP-qPCR
ACTIN11 CHIP-R	CCAAACGCAGAATAGCATGTGG	CHIP-qPCR
SPT-F (SfiI)	CGCGGATCCGGCCGTCAAGGCCATGATATCACAGAGAGAAGAAAGAGAAG	clone *SPT* coding sequence to pCambia1305
SPT-R (SfiI)	CGCGAATTCGGCCCATGAGGCCAAGTAATTCGATCTTTTAGGTCAGGTTG	
IND-F (SfiI)	CGCGGATCCGGCCGTCAAGGCCATGATGGAGCCTCAGCCTCACCATCTC	clone *IND* coding sequence to pCambia1305
IND-R (SfiI)	CGCGAATTCGGCCCATGAGGCCAGGGTTGGGAGTTGTGGTAATAACAAAGGTA	
HEC1-F (SfiI)	CGCGGATCCGGCCGTCAAGGCCATGGATTCTGACATAATGAA	clone *HEC1* coding sequence to pCambia1305
HEC1-R (SfiI)	CGCGAATTCGGCCCATGAGGCCATCTAAGAATCTGTGCAT	
EGFP F (XbaI)	CGAGGGATCCTCTAGAATGGTGAGCAAGGGCGAGG	clone the coding sequence of *EGFP*
EGFP R (PstI)	TCAAGCTTGCATGCCTGCAGTTACTTGTACAGCTCGTCCATGCC	
mRFP F (XbaI)	CGAGGGATCCTCTAGAATGGCCTCCTCCGAGGAC	clone the coding sequence of *mRFP*
mRFP R (PstI)	TCAAGCTTGCATGCCTGCAGTTAGGCGCCGGTGGAGTG	
SEC-F (SfiI)	CGCGGATCCGGCCGTCAAGGCCATGATCTCGTCCAAAAACGGAGCT	clone *SEC* coding sequence to pCambia1305
SEC-R (SfiI)	CGCGAATTCGGCCCATGAGGCCATCTGTCATGTGGGAATTCTAGGTC	
sec-2-R (SifI)	CGCGAATTCGGCCCATGAGGCCAAGTATTAACGATCTCAGGATC	Rev primer for cloning *SEC-2* in pCambia1305-3HA vector
SPY-F (SfiI)	CGCGGATCCGGCCGTCAAGGCCATGGTGGGACTGGAAGATGATACT	clone *SPY* or *SPY-3* coding sequence to pCambia1305
SPY-R (SfiI)	CGCGAATTCGGCCCATGAGGCCAGCTAGTGGAGTCCATTCTCTTTGA	

pPID F(PstI)	CCTCTAGAGTCGACCTGCAGGTGACCATGAGTTCTCTAGTTCAAC	clone the 1kb promoter of *PINOID* gene
pPID R(HindIII)	ACTGACCCATAAGCTTCGCCGGGAAAATCGAAGTTAAATC	
LB	GAACATCGGTCTCAATGCA	genotyping of *sec-2* mutant[32]
NS4R	AAACCAAGACAAAATCCAGATCCTCATCA	
GSP31	GCTCCGATCCAGGTTTCATA	
GSP33	ACACTTCGCCTGATATGTTCACTCTTC	
sec-5 LP	TCATGAATCAATCCTTGAGCC	genotyping of *sec-5* mutant[34]
sec-5 RP	TTTCGATGTCCCTTCTTTGTG	
SALK_LB1.3	ATTTTGCCGATTTCGGAAC	
spy-3 F	GCGACCTATCACCATTGGA	genotyping of *spy-3* mutant[35]
spy-3 R	AAAACAGTCCGGAAGCCTAACC	
SPY-NS1	CTCCTAAATGGCTGGACATAATTCAGATG	genotyping of *spy-4* mutant[35]
pSPY-1	CTAAAATCTTGTTCACCTTCAAAGAAACA	
SPY-insert	TCACTAAAGGCGGTAATACGGGTA	
spt-12 LP	AGACTCTAGAATTCGTGTCACC	genotyping of *spt-12* mutant[57]
spt-12 RP	GAAGAAGCAGAGAGTGATGGGAGA	
spt-12 LB	AACGTCCGCAATGTGTTATTAAGTTGTC	
I miR-s2 SEC	GATACGTTTTACAGGGGGCCCAGTCTCTCTTTTGTATTCC	amplify amiRNA sequence for *SEC*
II miR-a2 SEC	GACTGGGCCCCCTGTAAAACGTATCAAAGAGAATCAATGA	
III miR*s2 SEC	GACTAGGCCCCCTGTTAAACGTTTCACAGGTCGTGATATG	
IV miR*a2 SEC	GAAACGTTTAACAGGGGGCCTAGTCTACATATATATTCCT	
amiR-GG-F	TGAAGACTTAATGACAAACACACGCTCGGACG	Golden-Gate cloning of amiRNA sequence for *SEC*
amiR-GG-R	TGAAGACTTAAGCCATGGCGATGCCTTAAATAAAG	
5TPR-SEC-opti F	AACTCGAGCTCGCCGAATCATCCGCAGGCAAT	recombinant SEC protein expression
5TPR-SEC-opti R	ATGGCCGACGTCGACTTAGCGATCATGCGGAAATT	
3TPR-SPYopti-F	CCGGAATTCCCGCATTGTGCAGAAGCGTGTAATAAC	recombinant SPY protein expression
3TPR-SPYopti-R	ATGGCCGACGTCGACTTAGCTGGTGCTATCCATACGTTTGCTC	
10his-MBP-F	CCGCTCGAGAATGCATCATCATCATCATCATCATCATCATCATAAAATCGAAGAAGGTAAACTGG	clone the 10HIS-MBP sequence into pTrcHis vector
10his-MBP-R	CCCAAGCTTGCCTGCAGGTCGACTCTAGAGGA	
SPT F	CGCGGATCCGATGATATCACAGAGAGAA	clone *SPT* coding sequence for recombinant protein expression
SPT R	ATGCATTGGCTGCAGTCAAGTAATTCGATCTTT	
SPT S23A F	AGAGAGTGATGGGAGATAAGAAATTGATTGCATCTTCTTCTTCTTC	point mutation specific residues in SPT
SPT S23A R	GAAGAAGAAGAAGATGCAATCAATTTCTTATCTCCCATCACTCTCT	
S23-25A F	AGAGAGTGATGGGAGATAAGAAATTGATTGCAGCTGCTTCTTCTTCCTCGGT	
S23-25A R	ACCGAGGAAGAAGAAGCAGCTGCAATCAATTTCTTATCTCCCATCACTCTCT	

T71-74A F	CTTACTACTCCCCGGCGGCGGCTGCAGCGACGGCGTCTT TGATT	
T71-74A R	AATCAAAGACGCCGTCGCTGCAGCCGCCGCCGGGGAGT AGTAAG	
S60-61A F	CCGGCATATTTTCGACCGTGCTGCTCCTTTACCTTCTTACT A	
S60-61A R	TAGTAAGAAGGTAAAGGAGCAGCACGGTCGAAAATATG CCGG	
pgSPT-sYFP F (EcoRI)	CCGGAATTCCGAAGATTAAAATTAGTAACACTGA	clone the 5kb promoter of *SPT*
pSPT R (BspEI)	GGCCTTCCGGATACACCAACAACAAAAAAAAAGCAGT	
genomic SPT F (BspEI)	TGTTGGTGTATCCGGAATGATATCACAGAGAGAAGAAA GAG	infusion cloning primers for the 2.5kb genomic SPT-sYFP sequence
SPT-YFP R (KpnI)	GGCCTCACTGAGTGGGTACCTTACTTGTACAGCTCGTCC ATG	
SPTFL F GW	GGGGACAAGTTTGTACAAAAAAGCAGGCTTCATGATAT CACAGAGAGAAG	Y2H primers for domain analysis (Gateway)
SPTFL R GW	GGGGACCACTTTGTACAAGAAAGCTGGGTTCAAGTAATT CGATCTTTTAGGTCAGG	
SPT-N FWD	GGGGACAAGTTTGTACAAAAAAGCAGGCTTCATGATAT CACAGAGAGAAG	
SPT-N REV	GGGGACCACTTTGTACAAGAAAGCTGGGTAGCTTCATCA ACTACAGCTTC	
SPT-C FWD	GGGGACAAGTTTGTACAAAAAAGCAGGCTTCGGTAATA ATAACAACGTTCAAGG	
SPT-C REV	GGGGACCACTTTGTACAAGAAAGCTGGGTTCAAGTAATT CGATCTTTTAGGTCAGG	
SPT-AH FWD	GGGGACAAGTTTGTACAAAAAAGCAGGCTTCATGATAT CACAGAGAGAAG	
SPT-AH REV	GGGGACCACTTTGTACAAGAAAGCTGGGTACCACCGGA GAAACCAAAAC	
SEC-N FWD	GGGGACAAGTTTGTACAAAAAAGCAGGCTTCATGATCTC GTCCAAAAACGGAGC	
SEC-N REV	GGGGACCACTTTGTACAAGAAAGCTGGGTGAAGGGCTG GACACTTGGAAG	
SECFL FWD	GGGGACAAGTTTGTACAAAAAAGCAGGCTTCATGATCTC GTCCAAAAACGG	
SECFL REV	GGGGACCACTTTGTACAAGAAAGCTGGGTTTATCTGTCA TGTGGGAATTC	
SEC-C FWD	GGGGACAAGTTTGTACAAAAAAGCAGGCTTCTTCCATGC AATAGCATATCC	
SEC-C REV	GGGGACCACTTTGTACAAGAAAGCTGGGTTTATCTGTCA TGTGGGAATTCTAGG	
SPYFL FWD	GGGGACAAGTTTGTACAAAAAAGCAGGCTTCATGGTGG GACTGGAAGATG	
SPYFL REV	GGGGACCACTTTGTACAAGAAAGCTGGGTCTAGCTAGT GGAGTCCATTC	
SPY-N FWD	GGGGACAAGTTTGTACAAAAAAGCAGGCTTCATGGTGG GACTGGAAGATG	
SPY-N REV	GGGGACCACTTTGTACAAGAAAGCTGGGTTAATCTTGTG AAGCGCCAACCC	
SPY-C FWD	GGGGACAAGTTTGTACAAAAAAGCAGGCTTCCACCCTCA ATACACTTCATGGG	
SPY-C REV	GGGGACCACTTTGTACAAGAAAGCTGGGTCTAGCTAGT GGAGTCCATTC	
HEC1 FWD	GGGGACAAGTTTGTACAAAAAAGCAGGCTTCATGGATT CTGACATAATGAAC	

HEC1 REV	GGGGACCACTTTGTACAAGAAAGCTGGGTTCATCTAAGAATCTGTGCATTGC	
GFP-F (SpeI)	GGACTAGTATGGTGAGCAAGGGCGAGG	primers for cloning E*GFP*
GFP-R (BamHI)	CGCGGATCCCTTGTACAGCTCGTCCATGC	
RFP-F (XbaI)	CGAGGGATCCTCTAGAATGGCCTCCTCCGAGGAC	primers for cloning *RFP-NLS*
RFP-NLS-R (PstI)	CATTGGTTCTGCAGTTAAACTTTTCTTTTTTTTTAGGAGGTCCGGCGCCGGTGGAGTGGCGGC	
pPID-171-F	GCACCCCTATAATATGCCTAG	amplify the 171 bp *pPID* fragment
pPID-171-R	CAACGCGTGAGAGAGGT	
pPID-171-mut	GCACCCCTATAATATGCCTAGAGGAAATAAGAAGAAAAAATAATGGGGAAAAAGAGAAGCTACTGCTACACTTTCAGGTGATGAACAACGTCTCATGATGATCATATATCTTACGTCATCAAGTCCTCTTTTTCTCTCTTTCACTTTTCTGTTTACCTCTCTCACGCGTTG	synthesized 171 bp *pPID* fragment with two G-boxes mutated
