# Supplementary material for: O-glycosylation of the transcription factor SPATULA promotes style development in Arabidopsis
Source: Nat Plants. 2024 Jan 26;10(2):283–99. doi: 10.1038/s41477-023-01617-4 (PMC10881398; doi:10.1038/s41477-023-01617-4)
Supplement: Supplementary file 5 — Company names and catalogue numbers of commercial reagents used in this study. [file 41477_2023_1617_MOESM5_ESM.pdf]

Reagent	Company	catalog number
GFP-Trap® Magnetic Agarose	ChromoTek	gtma-20
MagRack™ 6, Cytiva	GE Healthcare	89129-096
Protein sample Buffer, Laemmli 2× Concentrate	Merck	S3401-10VL
TEAB buffer	Merck	15715-58-9
Sequencing Grade Modified Trypsin	Promega	V5111
pMAL-c2X® vector	Addgene	75286
pTrcHis-TRF2 vector	Addgene	50488
SfiI restriction enzyme	NEB	R0123S
DraIII restriction enzyme	NEB	R3510S
pDONR221® vector	Invitrogen	12536017
pGDAT7® and pGBKT7® vectors	Clontech	630489
35S::GVG:Nos cassette	Synbio TSL	pICSL11041
Ni-NTA Agarose	QIAGEN	30210
UDP-GlcNAc	Merck	U4375
GDP-fucose	Merck	G4401
Pierce™ Anti-HA Magnetic Beads	Thermo Scientific	88836
Erythrosin B	Sigma-Aldrich	198269
potassium iodide	Sigma-Aldrich	7681-11-0
Pierce™ D-Luciferin	Thermo Scientific	88291
Yeast Synthetic Drop-out medium (-L -W)	Sigma-Aldrich	Y0750-20G
Yeast Synthetic Drop-out medium (-L -W -H -A)	Sigma-Aldrich	Y2021-20G
3-AT (3-Amino-1,2,4-triazole)	Merck	A8056
Dexamethasone	Merck	D1756
DMSO	Merck	472301
6-benzylaminoadenine	Merck	B3408
RNeasy Plant Mini Kit	QIAGEN	74904
M-MLV Reverse Transcriptase	Promega	M1701
qPCRBIO SyGreen Blue Mix	PCRbio	PB20.15-01
GFP Monoclonal Antibody (*)	Thermo Scientific	GF28R
Biotin anti-RFP antibody (**)	abcam	ab34771
anti-mouse antibody (m-IgGκ BP-HRP) (***)	Santa Cruz	sc-516102
Goat Anti-Rabbit IgG H&L (HRP) (****)	abcam	ab205718
Anti-HA-Biotin (*****)	Merck	3F10
Monoclonal ANTI-FLAG® M2 antibody (******)	Merck	F3165

https://nph.onlinelibrary.wiley.com/doi/full/10.1111/nph.12364 *
https://www.nature.com/articles/s41467-020-19343-2 **
https://www.mdpi.com/1422-0067/21/24/9666 ***
https://bsppjournals.onlinelibrary.wiley.com/doi/full/10.1111/mpp.13037 ****
https://www.nature.com/articles/ncomms5848#Sec2 *****
https://www.nature.com/articles/s41477-022-01303-x ******
