# Supplementary figures and images for: O-glycosylation of the transcription factor SPATULA promotes style development in Arabidopsis
Source: Nat Plants. 2024 Jan 26;10(2):283–99. doi: 10.1038/s41477-023-01617-4 (PMC10881398; doi:10.1038/s41477-023-01617-4)

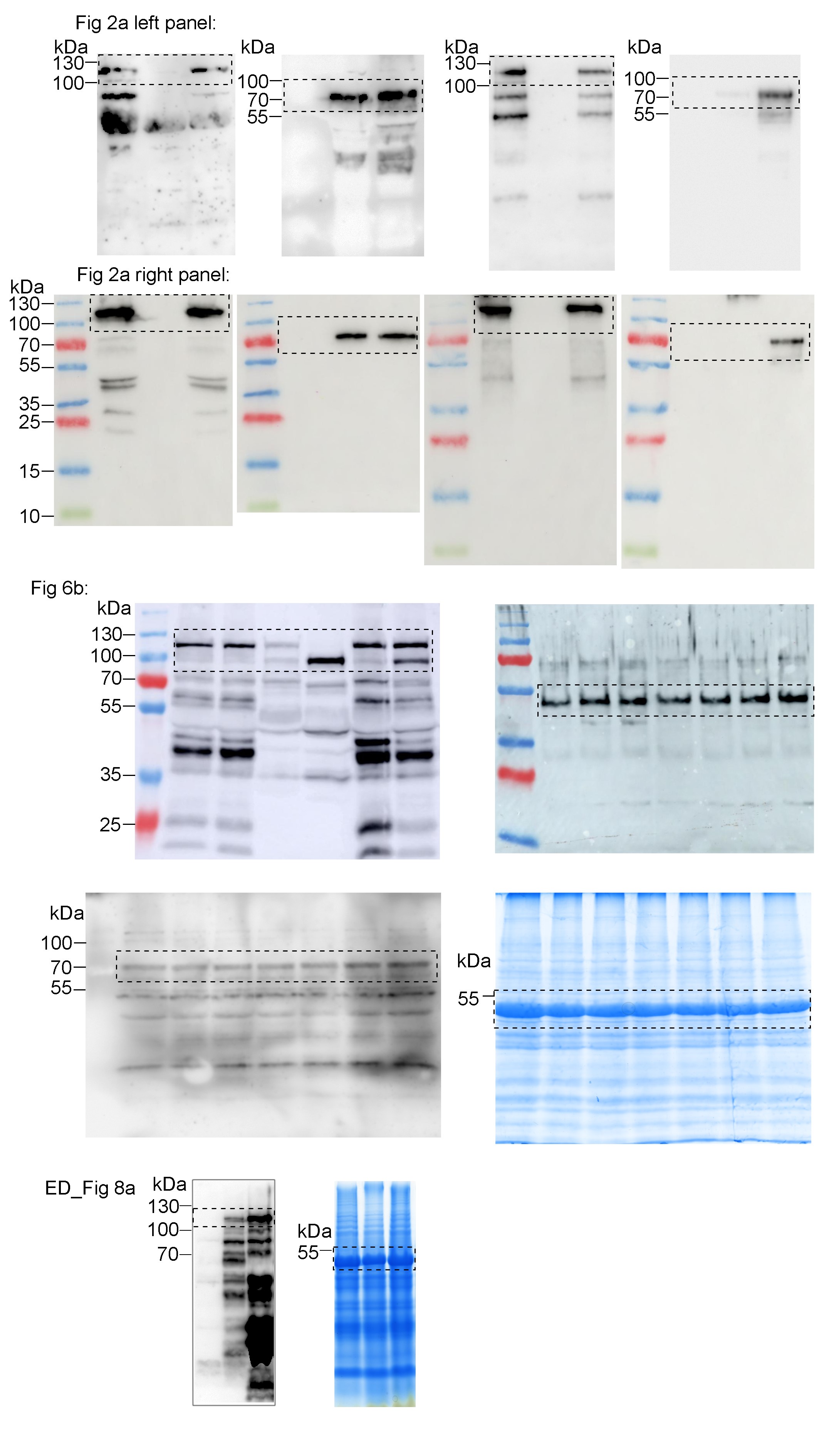

Supplement: Supplementary file 7 — Unprocessed western blots and/or gels for Figs. 2 and 6 and Extended Data Fig. 8. [file 41477_2023_1617_MOESM7_ESM.jpg]
